# Featured Immune Characteristics of Periodontitis and Primary Sjögren's Syndrome Revealed by Single‐Cell Transcriptome Analyses

**DOI:** 10.1111/jcmm.70713

**Published:** 2025-07-22

**Authors:** Juanjuan Li, Yuhui Chen, Chunfang Wang, Jinyang Yu, Qianyu Yang, Yan Liu, Chuhua Tang

**Affiliations:** ^1^ Department of Stomatology, Ninth Medical Center Chinese PLA General Hospital Beijing China; ^2^ Department of Stomatology Aviation General Hospital Beijing China; ^3^ Department of Traditional Chinese Medicine, Ninth Medical Center Chinese PLA General Hospital Beijing China

**Keywords:** cell communication, periodontitis, primary Sjögren's syndrome, scRNA

## Abstract

Periodontitis (PD) and primary Sjögren's syndrome (pSS) are persistent inflammatory diseases that affect oral health, and numerous studies have uncovered phenotypic and intrinsic correlations between the two. However, a comprehensive and high‐resolution analysis at the single‐cell level has not been reported comparing the immune cell landscapes in PD and pSS. This study integrated single‐cell transcriptome data from peripheral blood mononuclear cells (PBMCs) of healthy controls (HC), PD and pSS patients sourced from the NCBI and NGDC databases. The scRNA datasets were integrated, and cells were clustered using Seurat, with further subdivision of T/NK, myeloid cells and B cell subclusters. Next, NicheNetr was used to analyse the communication between myeloid cells and CD8^+^ naive T (Tn) cells. Finally, we identified nine T/NK cell subclusters, seven myeloid cell subclusters and five B cell subclusters. Compared to the HC group, the proportion of CD8^+^ Tn cells was significantly downregulated in both PD and pSS. HLA‐DRA^+^ mono cells showed a significant decrease in PD. Additionally, immature B cells were upregulated in PD but showed an opposite trend in pSS. The intracellular communication analysis between myeloid cells and CD8^+^ Tn cells suggested that myeloid cells may promote the exhaustion of CD8^+^ Tn cells through the *IL‐7/TNFSF11‐CDKN1A* signalling pathways. The *BACE2‐CDKN1A* signalling pathway may also contribute to the depletion of CD8^+^ Tn cells in both PD and pSS. Collectively, this study uncovers the single‐cell level associations between PD and pSS, providing new insights into the common molecular mechanisms underlying these two diseases.

## 1 Introduction

Periodontitis (PD) is a chronic multi‐factorial inflammatory disease that ranks as the sixth most prevalent chronic condition globally, affecting the oral health of over 11% of adults worldwide [1]. Plaque biofilm dysregulation is primarily the cause of the PD, which triggers a chronic inflammatory response in patients. This, in turn, leads to damage of periodontal tissues, including attachment loss, gingival recession and alveolar bone loss. In severe cases, PD can result in tooth loss and systemic inflammation, significantly impacting patients' chewing ability, nutritional status and quality of life [2, 3]. Primary Sjögren's syndrome (pSS) is a chronic inflammatory autoimmune disease that affects the oral cavity. It is characterised by lymphocytic infiltration of exocrine glands, particularly the salivary glands, which leads to reduced saliva secretion and subsequent oral dryness [4, 5]. Ninety percent of pSS patients are female, and a complex interaction of genetic and environmental factors may contribute to the development of pSS, but the specific aetiology remains uncertain [6, 7]. Studies have reported that oral dryness in pSS patients gives rise to a series of oral problems such as dental caries, oral candidosis, mucositis and dysphagia [8, 9]. Najera et al. previously demonstrated that the risk of PD in pSS patients is 2.2 times higher than that in healthy controls [10]. Conversely, epidemiological studies have indicated that patients with PD have an approximately 50% increased risk of developing pSS subsequently [11]. Lu et al. conducted a statistical analysis involving 1945 subjects and found that pSS patients had significantly higher utilisation rates of dental services for issues like dental caries and periodontitis in the 3 years before diagnosis compared to controls [12]. Conde et al. after analysing data from 15 paediatric SS patients also concluded that pSS patients have an increased risk of PD [13]. Following a comprehensive meta‐analysis of 21 studies, Bo et al. found that 5 studies identified a positive correlation between PD and pSS, while 16 studies indicated significantly poorer periodontal conditions in pSS patients versus controls [14]. Moreover, salivary biomarkers (e.g., IL‐4, IL‐6, IL‐10 and IL‐17) were elevated in both PD and pSS patients compared to controls [15], underscoring shared inflammatory pathways. These findings underscore a close association between PD and pSS. However, the molecular mechanisms underlying this association remain unclear. Therefore, exploring the shared genetic features of PD and pSS and their underlying molecular mechanisms holds great significance for diagnosing and treating PD and pSS.

Increasing clinical and experimental evidence suggests that inflammatory responses and immune cell infiltration play a crucial role in developing PD and pSS. PD is widely recognised as an inflammatory condition, often driven by the infiltration of sub‐gingival bacteria, which is exacerbated by pro‐inflammatory factors [16]. Interleukin (IL)‐6 is a key pro‐inflammatory marker in PD, significantly influencing bone resorption and osteoclast activation [17]. Studies have confirmed high levels of IL‐6 in both saliva and serum of pSS patients, and elevated IL‐6 levels have been found to correlate with the pSS condition [18, 19]. The IL‐17/Th17 axis activation has also been proven to regulate PD and pSS significantly [20, 21]. Furthermore, cytokines such as IL‐12, IL‐23 and T‐cell activation factor (CD44) are elevated in the saliva or serum of SS patients, contributing to low‐grade periodontal inflammation [22, 23]. Beyond cytokines, cellular characteristics indicate that in pSS patients, the infiltrating cells in salivary glands are predominantly CD4^+^ T cells and B cells. In the early stages of pSS, CD4^+^ T cell infiltration induces B cell activation through the production of proinflammatory cytokines, thereby participating in the pathogenesis of the pSS [24]. B cells are also recognised as a key factor in the pathogenesis of PD [25]. According to Kebschull et al., in aggressive PD, there is an activation of B cell receptor signalling and a 20% increase in the expression of the B cell surface marker CD19 compared to chronic PD [26]. Comparing patients with PD and pSS to those with PD but without pSS, Pers et al. found significantly higher levels of B‐cell activating factor (BAFF) in both serum and saliva of the PD and pSS cohort. Furthermore, these BAFF levels exhibited a positive correlation with the gingival index (GI). The authors suggest that B cells may negatively impact periodontal health in pSS patients by upregulating BAFF in their saliva [27]. These studies collectively hint at the intricate mechanisms underlying the close association between PD and pSS at the cellular and cytokine levels. However, as these investigations have focused on preselected specific cell types or factors, a comprehensive and high‐resolution analysis comparing the immune cell landscapes in PD and pSS remains essential to reveal their similarities and differences at the molecular level.

Single‐cell sequencing (scRNA‐seq), one of the most advanced techniques in current biomedical research, offers new insights into the diversity and functions of different cell types within a tissue or organism by sequencing the genetic material of individual cells [28]. Peripheral blood plays an integral role in the immune pathophysiology of PD and pSS, with peripheral blood mononuclear cells (PBMCs) serving as key immune cells. The single‐cell transcriptome profiling of PBMCs can provide a novel understanding of the molecular characteristics of immune cells in PD and pSS. Here, we collected and analysed scRNA datasets from PBMCs of patients with PD and pSS, mapped and compared the immune cell landscapes of the two diseases, offering new insights into their shared molecular mechanisms.

## 2 Methods

### 2.1 Dataset Collection

Single‐cell transcriptomic RNA (scRNA) sequencing data of PBMCs were collected from 14 PD patients, 8 pSS patients and 21 healthy controls (HC). These data were obtained from the NCBI (https://www.ncbi.nlm.nih.gov/) and NGDC (https://ngdc.cncb.ac.cn/?lang=zh) databases. The datasets included PRJNA660749 (HC, *n* = 5; pSS, *n* = 5) [29], HRA000916 (HC, *n* = 1; pSS, *n* = 3) [30], PRJNA1023400 (HC, *n* = 11; PD, *n* = 10) [31] and PRJNA730788 (HC, *n* = 4; PD, *n* = 4) [32]. Detailed information is provided in Table S1.

### 2.2 Quality Control and Analysis of scRNA‐Seq Data

The Cell Ranger software (v6.1.2, 10 × Genomics) [33] was utilised for aligning raw reads to the human reference genome GRCh38, filtering, barcode demultiplexing and unique molecular identifier (UMI) counting using the *count* command with default parameters. This process generated a feature‐barcode matrix for each sample. Seurat (v5.1.0) [34] was then employed to filter and integrate the gene‐barcode matrices from 46 samples. Cells were filtered based on the following criteria: (1) 200 < nFeature_RNA < 4500 and (2) percent.mt < 25%, resulting in 389,100 high‐quality cells (mean cells: HC, 9488; PD, 9859; pSS: 6459). Subsequently, data integration into a single dataset was performed using the Reciprocal PCA (rpca) algorithm of the *FindIntegrationAnchors* and *IntegrateData* functions. Before clustering and dimensionality reduction, data was processed using log normalisation and scaled for the top 2000 most variable genes using the *FindVariableFeatures* function. Based on the results of *PCElbowPlot*, the first 25 principal components were selected for uniform manifold approximation and projection (UMAP) using the *RunUMAP* function. After dimensionality reduction, cells were clustered using the *FindClusters* function with a resolution of 0.01. Subcluster segmentation was performed for T/NK, B, myeloid and megakaryocyte cells (MK) using resolutions of 0.05, 0.03, 0.09 and 0.01, respectively. Cell cluster annotation was based on the expression of classical marker genes.

### 2.3 Differentially Expressed Gene Identification

Differential expression analysis between different groups was conducted using the Seurat *FindMarkers* function. Genes with an adjusted *p*‐value < 0.05 and |log_2_FC| > 0.25 were defined as differentially expressed genes (DEGs).

### 2.4 Function Enrichment Analysis of DEGs

The clusterProfiler (v4.10.1) [35] or Metascape (http://metascape.org/gp/index.html#/main/step1) was employed to perform gene ontology (GO) and Kyoto Encyclopedia of Genes and Genomes (KEGG) enrichment analysis on the DEGs. The *gseKEGG* function from clusterProfiler was also used for gene‐set enrichment analysis (GSEA) of the genes. *p* value < 0.05 was considered a significant enrichment.

### 2.5 High‐Dimensional WGCNA Analysis

High‐Dimensional WGCNA (hdWGCNA) [36] is a tool for weighted gene co‐expression network analysis (WGCNA) of scRNAseq datasets. It aids in constructing cell type‐specific co‐expression networks and identifying interconnected genes within modules. This study used hdWGCNA to identify key modules associated with specific cell subclusters following a standard analysis pipeline. Subsequently, hub genes within this critical module were determined through screening.

### 2.6 Cell–Cell Interaction

The NicheNet (v2.2.0) [37] package was adopted to explore differential gene expression between disease and HC that may be caused by intracellular communication. NicheNet specifically predicts the priority of target genes in sender cells and their corresponding receiver cells, whose expression levels change during disease onset. Initially, DEGs within receiver cells between disease and HC were screened using the *FindMarkers* function based on criteria including an adjusted *p*‐value < 0.05, average log_2_FC > 0.25, and expression in more than 10% of receiver cells. Ligand activity analysis was conducted based on their target genes and default parameters. Ligands were ranked according to the Pearson correlation coefficient, and the top 10 ligands were selected for ligand‐target network analysis. Only ligands with regulatory potential scores greater than 0.33 were predicted to have target genes. Ligands and target genes were then used to infer potential signalling pathways between them. The predicted signalling pathways were visualised using Cytoscape (v3.10.2).

### 2.7 Statistical Analysis

The statistical methods and relevant thresholds for each analysis have been described in the aforementioned methodology section. Additionally, a comparative study of different subclusters was performed using a one‐way analysis of variance (ANOVA) with Tukey's post hoc test through SPSS 22.0 software (SPSS Inc., USA). *p* value < 0.05 was considered statistically significant.

## 3 Results

### 3.1 Global Landscape of the Immune Cell Composition of PD and pSS

**FIGURE 1:**
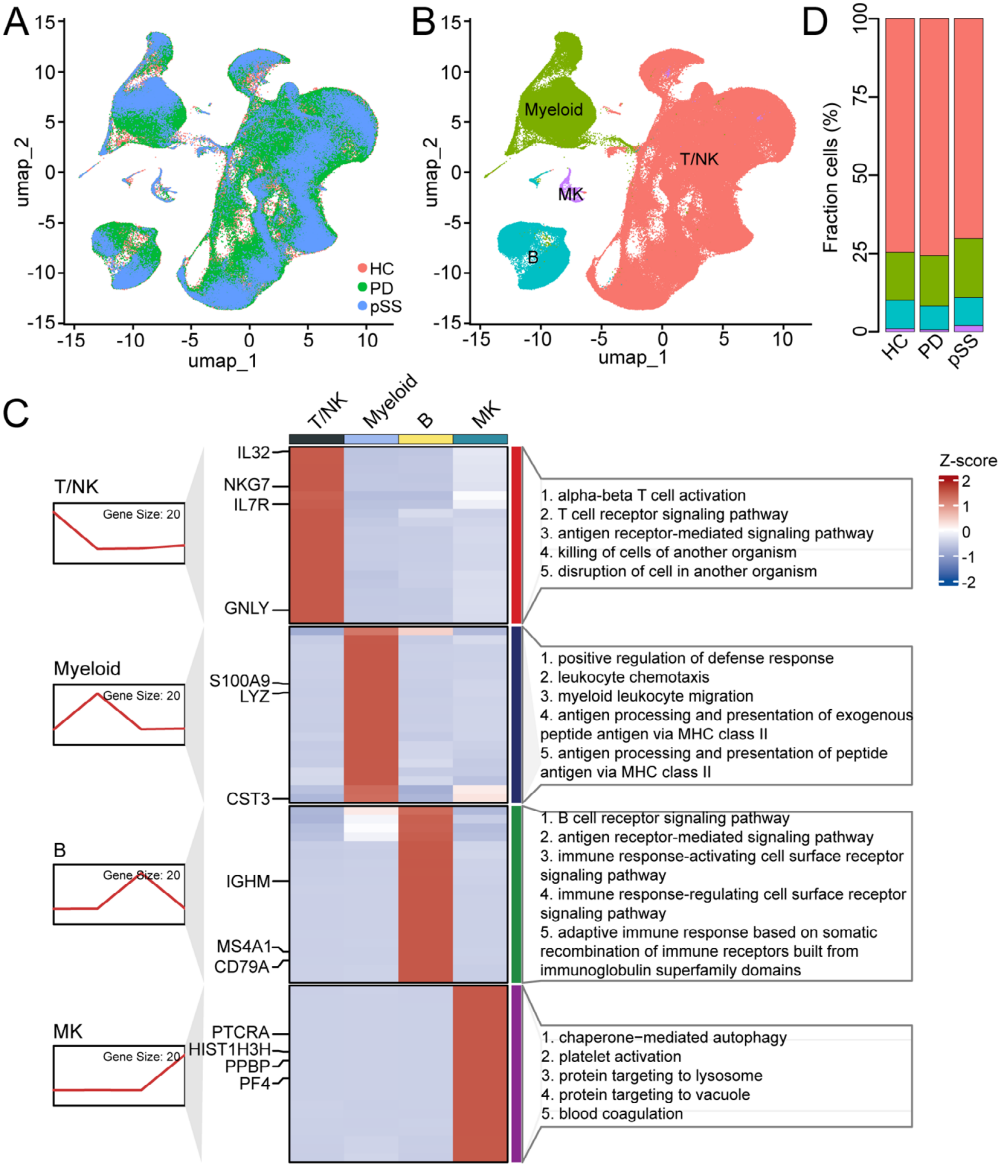
Global landscape of the immune cell composition of PD and pSS. (A) The UMAP plot shows HC, PD and pSS groups. (B) The same UMAP plot shows T/NK cells, myeloid cells, B cells and MK clusters. (C) ClusterGVis plot showing the top 20 marker genes in the 4 different cell clusters and the related GO enrichment terms based on those genes. (D) Bar plot showing the ratio of each cell type constitution in PD and pSS patients.

To investigate the similarities and differences in the immune cell landscapes of PD and pSS at the single‐cell level, we collected PBMC scRNA datasets of 14 PD patients, 8 pSS patients and 21 HCs. After rigorous quality control, all samples were integrated following batch effect correction (Figure 1A,B). Subsequently, 389,100 cells were subjected to further analysis (Table S2). Clustering was performed after dimensionality reduction using UMAP, resulting in four distinct cell clusters (Figure 1B). All four clusters consisted of cells from each sample, indicating effective batch effect correction (Figure 1A). These cell clusters were further annotated based on their characteristic marker genes as T/NK cells (*NKG7* ^+^ *GNLY* ^+^ *IL32* ^+^ *IL7R* ^+^), myeloid cells (*CST3* ^+^ *LYZ* ^+^ *S100A9* ^+^ *CD14* ^+^), B cells (*CD79A* ^+^ *CD79B* ^+^ *MS4A1* ^+^ *IGHM* ^+^) and megakaryocytes (MK, *PPBP* ^+^ *PF4* ^+^ *PTCRA* ^+^ *HIST1H3H* ^+^) (Figure 1D, Figure S1). Functional enrichment analysis of the top 20 marker genes for each of the four cell clusters revealed the following: T/NK cells are involved in alpha‐beta T cell activation and T cell receptor signalling pathways, myeloid cells participate in myeloid leukocyte migration, antigen processing, and presentation, B cells engage in the B cell receptor signalling pathway, and MKs are involved in platelet activation (Figure 1C). Next, we quantified the composition of immune cells in different disease states and HCs. Compared to the HC group, PD patients showed an increase in T/NK cells and a decrease in myeloid and B cells (Figure 1D, Table S2), while pSS patients exhibited a reduction in T/NK cells and an increase in myeloid and B cells. These findings suggest the presence of distinct immune microenvironments in the peripheral blood of these two diseases.

**FIGURE 2:**
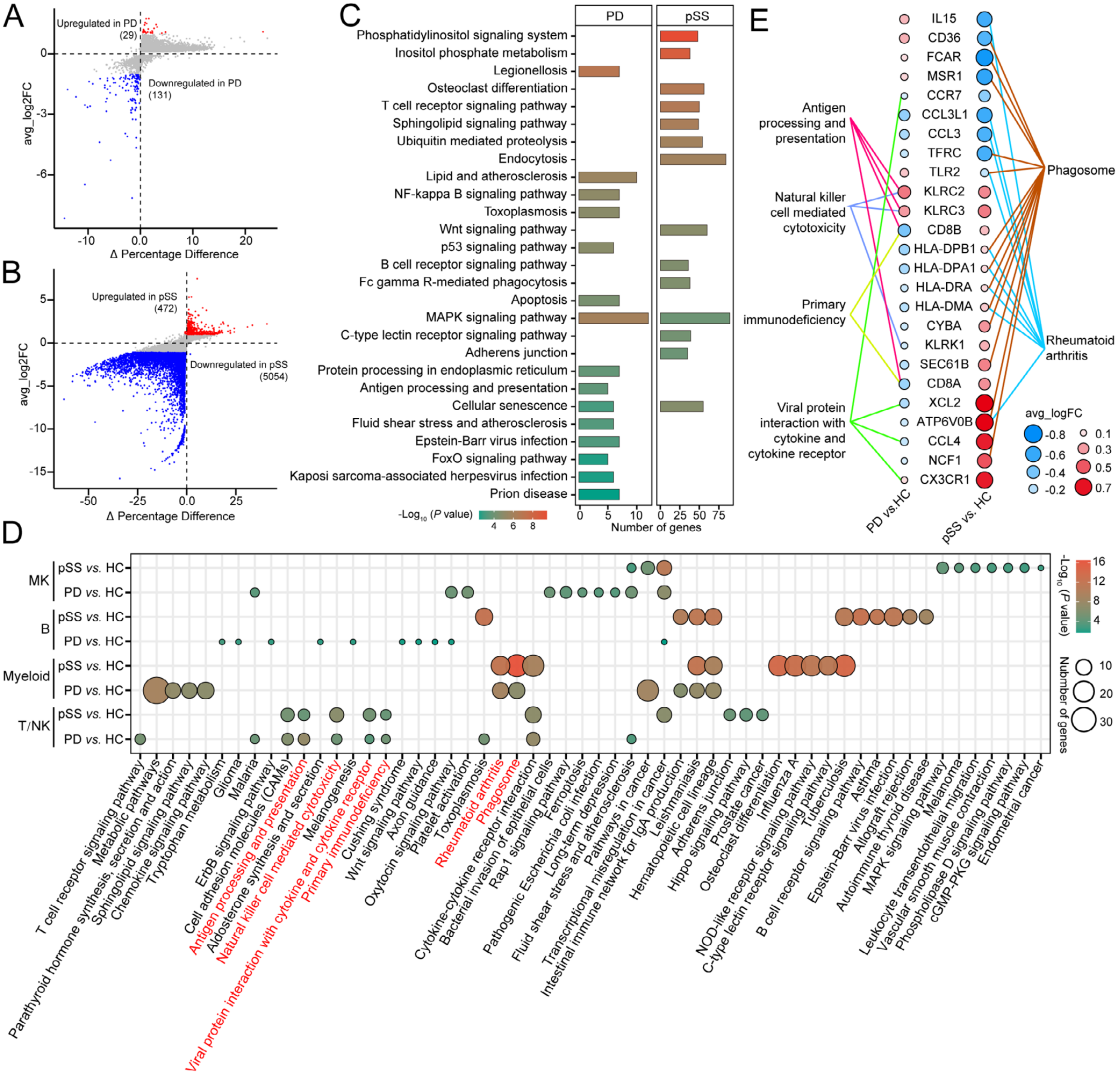
Pathway enrichment analysis in all PBMCs and cell clusters. (A, B) Volcano plot showing DEGs between PD (A) or pSS (B) patients with HCs in all PBMCs. (C) Pathway enrichment analysis of DEGs from (A) and (B). (D) Pathway enrichment analysis of DEGs between PD or pSS patients with HCs in different cell clusters. (E) Dot plots showed the average log_2_FC (avg_logFC) of key genes involved in the same pathway in PD and pSS.

To further dissect the molecular mechanisms underlying the pathogenesis of PD and pSS, we performed DEGs screening and functional enrichment analysis between disease and HC groups, aiming to discover the biological pathways altered in disease states from a global perspective. Compared to HC, PD showed 29 upregulated genes and 131 downregulated genes (Figure 2A, Table S3), while pSS exhibited 472 upregulated genes and 5054 downregulated genes (Figure 2B, Table S3). KEGG enrichment analysis of these DEGs revealed that PD primarily affects inflammatory‐related pathways such as the NF‐kappa B and p53 signalling pathways. In contrast, pSS mainly impacts pathways like the T cell receptor signalling pathway and endocytosis. Interestingly, both conditions affect the MAPK signalling pathway (Table S4). Considering that differential gene screening might influence functional enrichment, we conducted KEGG functional enrichment using GSEA for all genes. As shown in Figure S2A,B, the Hippo signalling pathway is activated in PD, while the NF‐kappa B signalling pathway and antigen processing and presentation are suppressed (Figure S2A). In pSS, the phenotype of systemic lupus erythematosus is significantly enriched, and salivary secretion and inflammatory mediator regulation of TRP channels are inhibited (Figure S2B). Similarly, the MAPK signalling pathway is suppressed in both PD and pSS. Compared to HC, the expression of genes related to the MAPK signalling pathway, such as *HSPA6*, *HSPA1A*, *HSPA1B*, *NR4A1* and *IL1RAP*, is also significantly reduced in PD and pSS (Figure S2C).

Different cell clusters often play distinct roles, prompting us to conduct functional analyses of DEGs within marker genes of various cell clusters. Compared to HC, 46 DEGs (37 upregulated and 9 downregulated) and 181 DEGs (71 upregulated and 110 downregulated) were identified from T/NK cell cluster marker genes in PD and pSS, respectively. Additionally, 334 (256 upregulated and 78 downregulated) and 550 (232 upregulated and 318 downregulated) genes were identified from myeloid cells in PD and pSS, respectively (Figure S3, Table S5). Functional enrichment analysis revealed that PD and pSS shared common signalling pathways in the T/NK cell cluster, such as antigen processing and presentation, natural killer cell‐mediated cytotoxicity and primary immunodeficiency. Similarly, rheumatoid arthritis (RA) and phagosome pathways were enriched in the myeloid cell cluster (Figure 2D, Table S6). Furthermore, analysis of genes within the same signalling pathways showed that although most genes exhibited opposite expression trends in PD and pSS, some genes displayed similar trends, including *CCR7*, *KLRC2*, *KLRC3* and *CX3CR1* (Figure 2E). Collectively, these results suggest that similar signalling pathways play crucial regulatory roles in both PD and pSS, albeit with specific gene‐level differences.

### 3.2 The CD8 ^+^ Tn Exhausted in PD and pSS

**FIGURE 3:**
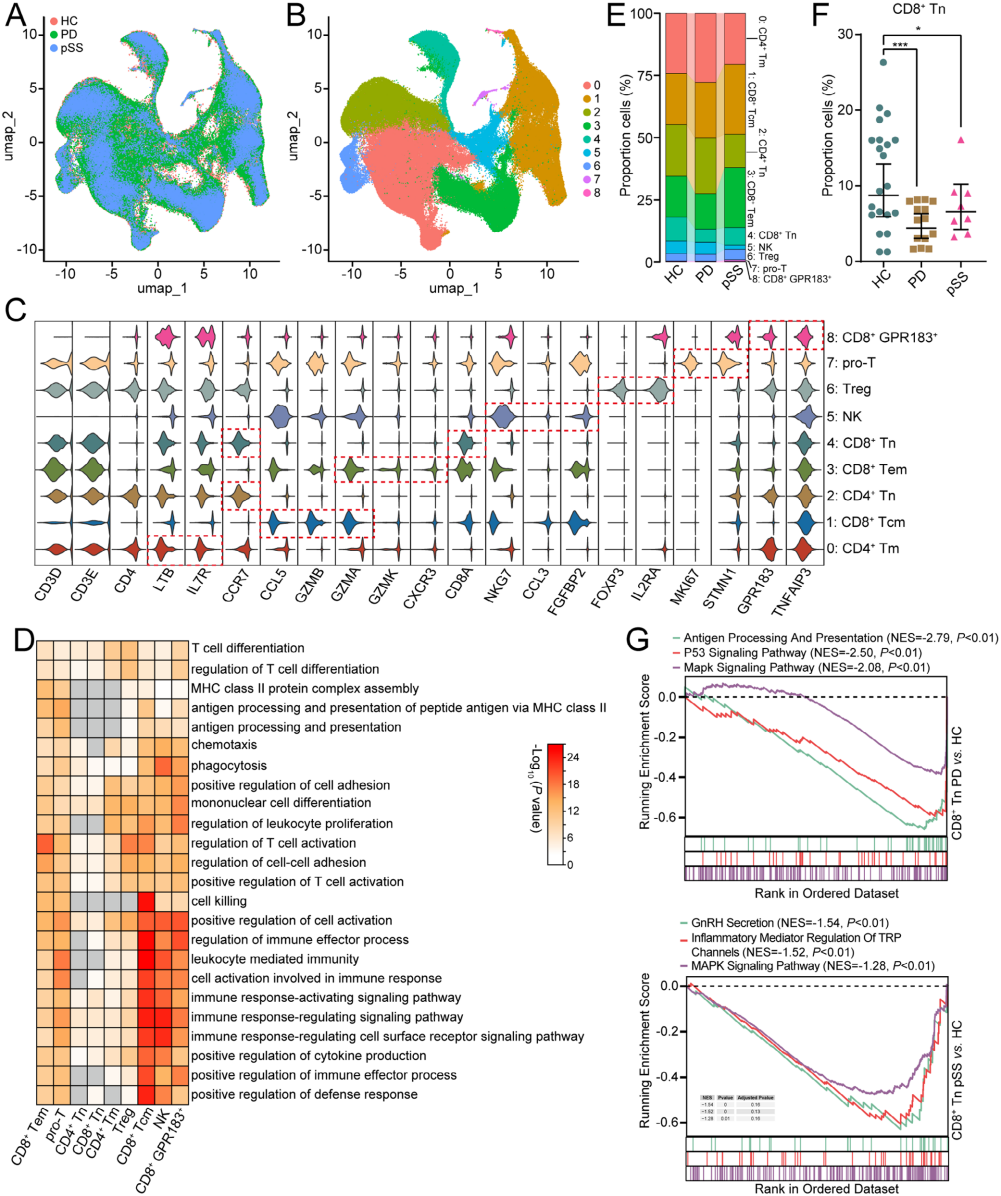
The CD8^+^ Tn is exhausted in PD and pSS. (A, B) UMAP plot showing T/NK subclusters profile with each cell coloured by different diseases (A) or associated cell types (B). (C) Multi‐violin plots showing the canonical marker genes for T/NK subclusters. (D) Enriched GO biological processes in each T/NK subcluster. (E) Bar plot showing each T/NK subcluster constitution ratio in PD and pSS patients. (F) The CD8^+^ Tn percent of T/NK cells in PD and pSS. (G) GSEA enrichment plot showing pathways enriched in PD or pSS CD8^+^ Tn versus HC CD8^+^ Tn.

T/NK cells represent a substantial cellular population that performs immunomodulatory functions. Therefore, we proceeded to re‐cluster T/NK cells to dissect their changes with finer resolution. Through UMAP dimensionality reduction followed by clustering, we obtained 9 subclusters (Figure 3A,B). Based on conventional cell marker genes, these cellular groups were classified as CD4^+^ T cell subtypes (*CD3D* ^+^ *CD3E* ^+^ *CD4* ^+^), including CD4^+^ Tm (*LTB* ^+^ *IL7R* ^+^), CD4^+^ Tn (*CCR7* ^+^) and Treg cells (*FOXP3* ^+^ *IL2RA* ^+^); CD8^+^ T cell subtypes (*CD8A* ^+^), comprising CD8^+^ Tcm (*CCL5* ^+^ *GZMB* ^+^ *GZMA* ^+^), CD8^+^ Tem (*GZMA* ^+^ *GZMK* ^+^ *CXCR3* ^+^), CD8^+^ Tn (*CCR7* ^+^) and CD8^+^ GRP183^+^ cells (*GRP183* ^+^ *TNFAIP3* ^+^); pro‐T cells (*MKI67* ^+^ *STMN1* ^+^); and NK cells (*NKG7* ^+^ *CCL3* ^+^ *FGFBP2* ^+^) (Figure 3C). To gain further insights into the functions of each subcluster, we conducted GO analysis. CD8^+^ Tem, CD8^+^ Tcm and Treg cells regulated T cell activation and cell–cell adhesion. CD4^+^ Tn and CD8^+^ Tn participated in T cell differentiation and mononuclear cell differentiation. CD8^+^ Tcm, CD8^+^ GRP183^+^ and NK cells were implicated in cell‐killing and immune response‐activating signalling pathways (Figure 3D).

Next, we compared different T/NK cell ratios (Figure 3E, Table S7). Compared to the HC group, the proportion of CD8^+^ Tcm cells showed an increasing trend in both PD and pSS. Conversely, the proportion of CD8^+^ Tn cells decreased significantly, indicating an immunosuppressive state of the CD8^+^ Tn cell subcluster (Figure 3F). Additionally, compared to the HC group, the proportions of CD4^+^ Tm, CD4^+^ Tn and NK cells were significantly reduced in pSS, while these cell ratios exhibited an upward trend in PD. Further GSEA functional enrichment analysis of genes in CD8^+^ Tn cells from PD and pSS revealed distinct patterns. Compared to HC, the antigen processing, presentation and p53 signalling pathways were significantly suppressed in PD. In pSS, the GnRH secretion pathway and inflammatory mediator regulation of TRP channels were notably inhibited, while the MAPK signalling pathway was suppressed in both diseases (Figure 3G).

Finally, we applied hdWGCNA to analyse the T/NK cell cluster dataset to identify genes associated with CD8^+^ Tn. The dendrogram identified four modules (M1–M4) (Figure S4A), among which M2, M3 and M4 were significantly depleted in CD8^+^ Tn (Figure S4B). Functional enrichment analysis of each module's top 30 hub genes revealed that M2 was significantly enriched in genes related to the MAPK signalling pathway, negative cell cycle regulation, TCR signalling modulators and T‐cell activation. Compared to HC, key genes such as *HSPA1A/B*, *DUSP1/2*, *NR4A1/2* and *NFKB1A* were significantly downregulated in both PD and pSS (Figure S4C–E). These results indicate that inactivation of signalling pathways (e.g., MAPK) contributes to CD8^+^ Tn depletion, subsequently driving immune dysfunction in PD and pSS.

### 3.3 The HLA‐DRA ^+^ Mono Exhausted in PD

**FIGURE 4:**
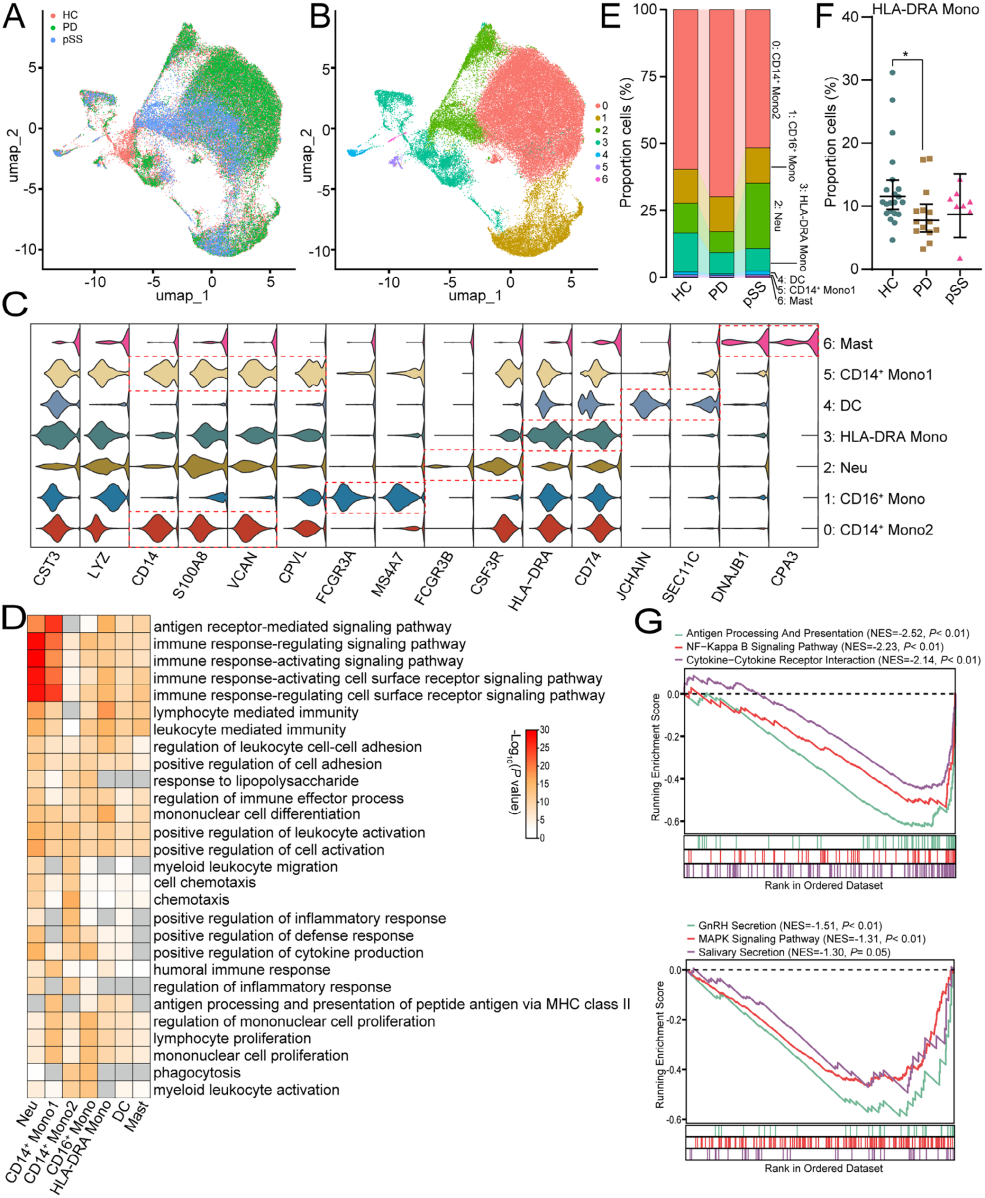
The HLA‐DRA^+^ Mono exhausted in PD. (A, B) UMAP plot showing myeloid subclusters profile with each cell coloured by different diseases (A) or associated cell types (B). (C) Multi‐violin plots showing the canonical marker genes for myeloid subclusters. (D) Enriched GO biological processes in each myeloid subcluster. (E) Bar plot showing the ratio of each myeloid subcluster constitution in PD and pSS patients. (F) The HLA‐DRA^+^ Mono percent of T/NK cells in PD and pSS. (G) GSEA enrichment plot showing pathways enriched in PD or pSS HLA‐DRA^+^ Mono versus HC HLA‐DRA^+^ Mono.

Myeloid cells, the second largest population in PBMCs, play a pivotal role in the pathogenesis of both PD and pSS. To further explore the similarities and differences between the immune cells of PD and pSS, we re‐clustered the myeloid cells. We identified seven myeloid cell subclusters through UMAP dimensionality reduction followed by clustering (Figure 4A,B). Based on their characteristic cell marker genes, these clusters were defined as CD14^+^ Mono1 (*CD14* ^+^ *S100A8* ^+^ *VCAN* ^+^), CD14^+^ Mono2 (*CD14* ^+^ *S100A8* ^+^ *CPVL* ^+^), CD16^+^ Mono (*FCGR3A* ^+^ *MS4A7* ^++^), HLA‐DRA^+^ Mono (*HLA‐DRA* ^+^ *CD74* ^+^), neutrophil (Neu, *FCGR3B* ^+^ *CSF3R* ^+^), dendritic cell (DC, *JCHAIN* ^+^ *SEC11C* ^+^) and mast cell (*DNAJB1* ^+^ *CPA3* ^+^) (Figure 4C). GO analysis revealed that almost all cell subtypes were involved in immune response‐regulating signalling pathways and lymphocyte‐mediated immunity (Figure 4D), indicating an activated immune state of the myeloid cells. Additionally, nearly all cell subtypes participated in the positive regulation of cell activation and lymphocyte proliferation. CD14^+^ Mono2 and CD16^+^ Mono were also predominantly involved in processes such as phagocytosis, suggesting that myeloid cells, mainly monocytes, may regulate lymphocyte proliferation and apoptosis.

Next, we compared the proportions of different myeloid cells (Figure 4E, Table S8). Compared to the HC group, the proportion of HLA‐DRA^+^ Mono cells showed a decreasing trend in pSS and a significant decrease in PD (Figure 4F). The proportion of DC cells increased significantly in pSS but showed an opposite trend in PD. We further performed GSEA functional enrichment on genes in HLA‐DRA^+^ Mono cells from PD and pSS samples. The results indicated that, compared to HC, antigen processing and presentation, NF‐Kappa B signalling pathway and cytokine‐cytokine receptor interaction were significantly suppressed in PD. In pSS, GnRH, salivary and MAPK signalling pathways were inhibited considerably (Figure 4G).

Finally, we employed hdWGCNA to analyse the myeloid cell cluster dataset to identify genes associated with HLA‐DRA^+^ Mono. The dendrogram identified nine modules (M1‐M9) (Figure S5A), among which M1, M4, M5, M6 and M7 were significantly enriched in HLA‐DRA^+^ Mono (Figure S5B). Functional enrichment analysis of each module's top 30 hub genes revealed that M7 was significantly enriched for MHC class II protein complex assembly genes. However, compared to HC, some key genes such as *HLA‐DRB1*, *CSF2RA*, *FLT3*, *CD86* and *NEGR1* showed opposite expression trends in PD and pSS (Figure S4C–E), suggesting that despite the decreasing trend of HLA‐DRA^+^ Mono in both PD and pSS, their roles may still differ.

### 3.4 B Cells Displayed Dysregulation in PD and pSS

**FIGURE 5:**
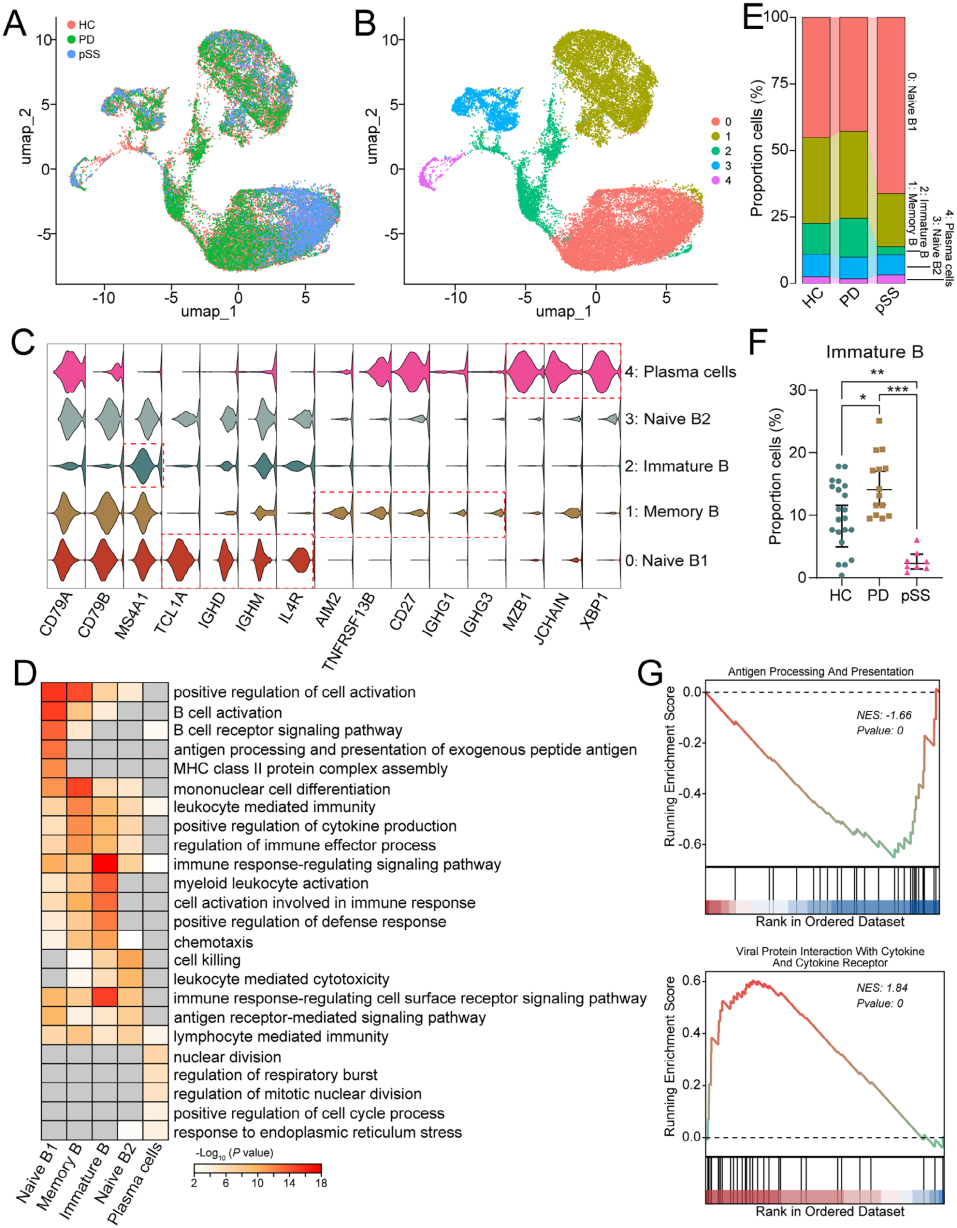
B cells displayed dysregulation in PD and pSS. (A‐B) UMAP plot showing B cell subclusters profile with each cell coloured by different diseases (A) or associated cell types (B). (C) Multi‐violin plots showing the canonical marker genes for B cell subclusters. (D) Enriched GO biological processes in each B cell subcluster. (E) Bar plot showing the ratio of each B cell subcluster constitution in PD and pSS patients. (F) The immature B percent of T/NK cells in PD and pSS. (G) GSEA enrichment plot showing pathways enriched in PD or pSS immature B versus HC immature B.

B cells exhibit opposing changes in PD and pSS, but the specific cell types involved remain unclear. Therefore, we also conducted a more refined classification of B cells. Through UMAP dimensionality reduction and clustering, B cells were regrouped into five clusters (Figure 5A,B). Based on their typical cellular marker genes, these clusters were defined as naive B1/2 (*TCL1A* ^+^ *IGHD* ^+^ *IGHM* ^+^ *IL4R* ^+^), immature B (*MS4A1* ^+^), memory B (*AIM2* ^+^ *TNFRSF13B* ^+^ *CD27* ^+^ *IGHG1/3* ^+^) and plasma cells (*MZB1* ^+^ *JCHAIN* ^+^ *XBP1* ^+^) (Figure 5C). GO analysis revealed the specialisation of B cell subtypes into different functions: naive B1 is involved in regulating B cell activation and the B cell receptor signalling pathway; memory B participates in mononuclear cell differentiation and leukocyte‐mediated immunity; and immature B is involved in the immune response‐regulating signalling pathway (Figure 5D). Next, we conducted a comparative analysis of the proportions of different B cell subclusters (Figure 5E, Table S9). Compared to the HC, the proportion of naive B1 increased in PD, while the proportion of immature B decreased. Conversely, in pSS, the proportion of immature B increased, while the proportion of memory B decreased (Figure 5F). Further GSEA functional enrichment analysis of genes in immature B cells revealed significant inhibition of antigen processing and presentation in PD compared to HC. In pSS, viral protein interaction with cytokines and cytokine receptors was significantly activated (Figure 5G). Using hdWGCNA to analyse the B cell cluster dataset, we identified three modules (Figure S6A), with M1 significantly enriched in immature B (Figure S6B). Functional enrichment analysis of each module's top 30 hub genes revealed that M1 was significantly enriched in genes that regulate the B cell receptor signalling pathway. Simultaneously, compared to HC, key genes *PRKCE*, *CAMK1D* and *BANK1* showed opposing expression trends in PD and pSS (Figure S5C–E), suggesting different alterations and regulatory roles of immature B in PD and pSS.

### 3.5 Myeloid Cells Promote the Depletion of CD8 ^+^ Tn Cells by Targeting CDKN1A

Myeloid subpopulations (e.g., monocytes) are established regulators of T cell proliferation and apoptosis [38, 39]. Simultaneously, studies have indicated that myeloid‐derived suppressor cells (MDSCs) can negatively regulate T cells, exerting an immunosuppressive effect [40]. Therefore, we subsequently employed NicheNet to explore the intracellular interactions between myeloid cells and T cells, investigating whether myeloid cells influence T cell suppression. We selected all types of myeloid cells as sender cells since they are generally involved in lymphocyte proliferation and positive regulation of cell activation (Figure 4D). CD8^+^ Tn cells were chosen as receiver cells due to the significant decrease in their proportion in both PD and pSS (Figure 4F).

**FIGURE 6:**
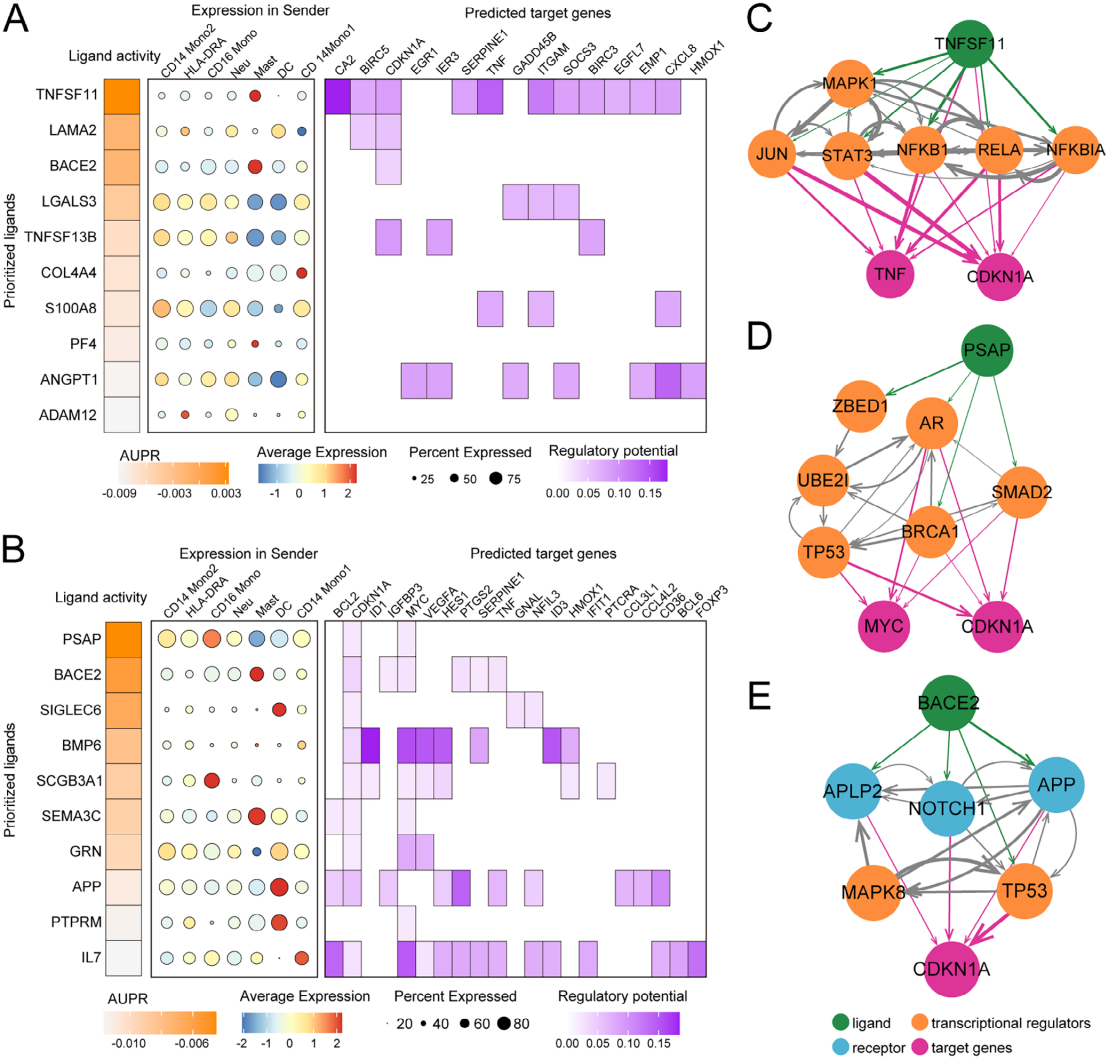
Myeloid cells might facilitate the exhaustion of CD8^+^ Tn cells via targeting CDKN1A. (A) Top 10 ligands identified in PD versus HC (left), the expression of ligands in myeloid cells (middle) and the interacting target genes in CD8^+^ Tn (right) predicted by NicheNet. (B) Top 10 ligands identified in pSS versus HC (left), the expression of ligands in myeloid cells (middle) and the interacting target genes in CD8^+^ Tn (right) predicted by NicheNet. (C–E) Network of the potential signalling pathways between the ligand TNFSF11 (C), PSAP (D), BACE2 (E), and their predicted target genes, respectively.

Compared to HC, we constructed interaction networks between myeloid cells and CD8^+^ Tn cells in PD and pSS, respectively. In PD, the ligand‐target interactions were primarily focused on the ligand *TNFSF11* (Figure 6A). *TNFSF11* is predominantly expressed in mast cells and regulates *TNF* and *CDKN1A* through transcriptional regulators such as *STAT3*, *NFKB1*, *MAPK1* and *JUN* (Figure 6C). In contrast to PD, ligand‐target interactions were denser in pSS (Figure 6B). Among them, the *PSAP* ligand was highly expressed in all subtypes of myeloid cells and modulated *MYC* and *CDK1A* via *AR*, *SMAD2*, *TP53*, etc. (Figure 6D). Additionally, in pSS, *IL7* played a crucial regulatory role by targeting numerous genes. For instance, it regulated *BCL2*, *MYC* and *FXOP3* through *IL7R*, *IL2RG* and *STAT*s (Figure S7). Interestingly, we observed high ligand activity of *BACE2* in both PD and pSS (Figure 6A,B). *BACE2* is primarily expressed in mast cells and regulates *CDKN1A* via *NOTCH1*, *MAPK8* and *TP53* (Figure 6E). This suggests that the IL7/TNSF11A/BACE2‐CDKN1A signalling pathway may be a key pathway through which myeloid cells promote the rapid depletion of CD8^+^ Tn cells in both PD and pSS.

## 4 Discussion

Both PD and pSS threaten human oral health, and numerous studies have indicated phenotypic and intrinsic connections between them [19, 27]. By depicting and comparing the immune cell landscapes of PD and pSS at the single‐cell level, we can deepen our understanding of these diseases' molecular mechanisms and gain novel perspectives for improved treatments.

In this study, we observed a significant decrease in the proportion of CD8^+^ Tn cells in both PD and pSS compared to HC. CD8^+^ Tn cells originate in the bone marrow, mature in the thymus, and circulate between secondary lymphoid organs via the blood and lymphatic systems [41]. During pathogenic infections, resting CD8^+^ Tn cells undergo proliferation and effector differentiation upon activation by specific antigens [42]. However, when exposed to continuous antigenic stimulation, as occurs in persistent inflammatory conditions like PD and pSS, CD8^+^ Tn cells may undergo exhaustion, thereby weakening their response to subsequent antigenic challenges [43, 44]. Madara et al. reported that PD patients had a lower proportion of CD8^+^ Tn cells at baseline compared to healthy controls. However, they observed a significant recovery in these cells at 6 and 12 months after treatment through flow cytometry analysis of PBMCs from 54 PD patients [45]. In pSS, Hong et al. found a lower proportion of CD8^+^ Tn cells compared to healthy controls using single‐cell analysis [29]. Conversely, Kudryavtsev et al. observed a significantly higher proportion and number of CD8^+^ Tn cells through flow cytometry [46]. These discrepancies may be related to different stages of the disease. Besides T cells, B cells also play a crucial role in PD and pSS. Our study found that the proportion of immature B cells was increased in PD but decreased in pSS compared to HC. This may be associated with the high expression of BAFF in pSS, which induces the transition of immature B cells to mature B cells [27]. In PD, Demoersman et al. reported a decrease in the proportion of mature B cells in the gingival tissues of patients with severe periodontitis compared to healthy controls. Additionally, in peripheral blood, there was a decrease in the proportions of B1 cells (*CD20* ^+^ *CD69* ^*−*^ *CD43* ^+^ *CD27* ^+^ *CD11b* ^+^) and *CD11b* ^+^ *CD5* ^+^ B cells, while other B cell subsets remained unchanged. This may indirectly support the upregulation of immature B cell proportion in PD [47]. Furthermore, compared to HC, the proportion of HLA‐DRA^+^ Mono cells was significantly decreased in PD, which may contribute to the attenuation of antigen presentation in this disease [48].

CD8^+^ Tn cells are regulated by various cytokines, including the γ chain (γc) family cytokines, such as IL‐2, IL‐4, IL‐7 and IL‐15 [49, 50]. In particular, IL‐7, along with its cognate ligand, plays a crucial role in the survival of CD8^+^ Tn cells. Persistent IL‐7 signalling may differentially affect the phenotype and function of CD8^+^ Tn cells. Some studies suggest that this could be attributed to the differential expression of IL‐7 receptor (IL‐7R) in CD8^+^ Tn cells [51]. Desvaux et al. demonstrated high expression of IL‐7R in epithelial cells and CD4^+^ and CD8^+^ T cells in salivary gland tissue. Moreover, compared to non‐pSS patients, pSS patients showed increased IL‐7R expression and activation of the IL‐7 signalling pathway [52]. Liang et al. also found elevated IL‐7 expression in the salivary glands, serum and saliva of pSS patients, accompanied by increased IL‐7R expression in the salivary glands but decreased expression in peripheral blood [53]. However, our study showed no significant increase in IL‐7R gene expression in pSS CD8^+^ Tn cells compared to HC (avg_log_2_FC = 0.01, *p* = 0.916). This may align with the broader view that the impact of IL‐7 on CD8^+^ T cells could be due to intrinsic self‐reactivity differences rather than cytokine receptor expression. CD5^high^ cells within the CD8^+^ Tn population respond more strongly to IL‐7 than CD5^low^ cells [54]. Treating T cells isolated from the synovial fluid of RA patients with anti‐CD5 monoclonal antibodies notably suppresses their proliferation when induced by IL‐2 [55]. Our data showed increased CD5 expression in pSS CD8^+^ Tn cells compared to HC, although the difference was insignificant (avg_log_2_FC = 0.709, *p* = 0.309). Therefore, the heterogeneity of CD8^+^ Tn cells in disease states may lead to differential responses to IL‐7 signalling, which requires further investigation in pSS. Additionally, our findings highlight the expression of PSAP in various myeloid cell subsets, where it exhibits the highest ligand activity. PSAP can target the breast and ovarian cancer susceptibility protein 1 (BRCA1) and subsequently affect CDKN1A, leading to the depletion of CD8^+^ Tn cells. This mechanism potentially contributes to the higher female incidence of pSS.

Among non‐γc family cytokines, TNF has garnered significant attention. Costimulatory signals initiated by TNF ligand‐receptor superfamily interactions promote the clonal expansion, differentiation, and survival of antigen‐triggered CD4^+^ and CD8^+^ T cells, playing a crucial role in T cell‐mediated adaptive immunity and disease pathogenesis [56]. Elevated plasma levels of TNFSF11 (RANKL), a TNF superfamily member, are reported in PD patients compared to HC and correlated with clinical attachment loss [57]. This likely relates to TNFSF11's role in stimulating osteoclast differentiation from precursors, increasing bone resorption [58]. Our study identifies a potential role for mast cells in regulating CD8^+^ Tn cells in PD via the TNFSF11 signalling pathway. While direct evidence is lacking, mast cells are known to enhance T cell proliferation and activation through OX40L (another TNF superfamily member) [59, 60]. BACE2, highly expressed in mast cells, appears to regulate CD8^+^ Tn cells in both PD and pSS. Primarily expressed in granulocytes derived from myeloid precursors, our findings confirmed BACE2 expression in all myeloid cells. However, current research on BACE2 focuses largely on its role in neurodegenerative diseases, where it inhibits the production of Aβ42 peptide in Alzheimer's disease [61]. Its specific functions in PD and pSS require further validation. Furthermore, myeloid‐derived suppressor cells (MDSCs), a heterogeneous population arising from immature myeloid cells under chronic inflammation, can infiltrate inflammatory sites. They inhibit the proliferation and migration of CD4^+^ and CD8^+^ T cells and promote their apoptosis [62]. Our study found that MDSCs (*CD33* ^+^ *CD11B* ^+^ *CD14* ^*−*^ *HLA‐DRA* ^*−*^) concentrated within mast cell populations (Figure S8), suggesting their potential contribution to CD8^+^ Tn cell exhaustion. These findings collectively suggest that blocking PSAP and TNFSF superfamily signalling may restore the proportions of CD8^+^ Tn cells and B cells, thereby offering therapeutic potential for pSS and PD. Existing research provides some support: PSAP reduces the proportion of CD8^+^ T cells in PBMC [63], and hydroxychloroquine (HCQ), an immunomodulatory drug used for RA and systemic lupus erythematosus (SLE), binds to saposin B (a cleavage product of PSAP) [64]. However, whether HCQ promotes CD8^+^ Tn cell recovery specifically via PSAP targeting requires verification. Similarly, TNFSF13B (affecting B cell maturation and development [65]) silencing has shown therapeutic efficacy in models like triple‐negative breast cancer [66], supporting the broader strategy of targeting these pathways.

Although NicheNetr implicated myeloid cells (including DCs) in CD8^+^ Tn regulation via ligands like TNFSF11/BACE2‐CDKN1A, these predictions require experimental validation. Notably, CDKN1A's role in T cell suppression remains unconfirmed, and sender‐receiver analysis may lack precision due to heterogeneous myeloid subsets. However, whether the mechanism underlying CD8^+^ Tn cell depletion is similar to that of T cell depletion in other chronic inflammatory diseases remains to be further investigated.

Apart from the changes in the proportion of immune cells, we also observed alterations in signalling pathways in both PD and pSS. For instance, in PD, antigen processing and presentation suppression were evident in various cell subclusters, including PBMCs, CD8^+^ Tn cells and HLA‐DRA^+^ Mono cells. This finding aligns with the phenotype characterised by a decrease in DC [67] and HLA‐DRA^+^ mono cells, suggesting that the attenuation of antigen presentation contributes to the progression of PD. In pSS, salivary secretion and inflammatory mediator regulation of TRP channels were inhibited, consistent with the phenotype of pSS [68]. The MAPK signalling pathway also underwent significant suppression in both PD and pSS. Current research suggests that the activation of MAPK promotes the development of PD and pSS while inhibiting the MAPK signalling pathway can ameliorate the phenotypes of PD [69] and pSS [70]. However, these studies were conducted using samples from gingival or salivary gland tissues, which may not fully align with the results obtained from PBMCs, necessitating further validation. Furthermore, among the commonly downregulated genes related to the MAPK pathway, *NR4A1* and *GADD45A* are involved in regulating the immune function of macrophages, T cells and other immune cells [71, 72], indicating the significant role of MAPK in immune modulation in PD and pSS.

## 5 Conclusion

In summary, we analysed the similarities and differences between PD and pSS at the single‐cell level. The proportions of CD8^+^ Tn cells and LHA‐DRA^+^ mono cells decreased in both PD and pSS, while the proportion of immature B cells increased in PD but decreased in pSS. Through analysis of intracellular communication, we found that *IL‐7/TNFSF11–CDKN1A* signalling pathways may regulate the exhaustion of CD8^+^ Tn in PD and pSS, respectively. Additionally, *BACE2–CDKN1A* may play a role in regulating CD8^+^ Tn in both PD and pSS. Overall, this study uncovers cellular and molecular associations between PD and pSS, providing new insights into the common molecular mechanisms of these two diseases.

## Author Contributions

**Juanjuan Li:** conceptualization (equal), methodology (equal), resources (equal), writing – original draft (equal). **Yuhui Chen:** data curation (equal). **Chunfang Wang:** data curation (supporting). **Jinyang Yu:** methodology (equal). **Qianyu Yang:** methodology (equal). **Yan Liu:** writing – review and editing (equal). **Chuhua Tang:** conceptualization (equal), project administration (equal), writing – review and editing (equal).

## Conflicts of Interest

The authors declare no conflicts of interest.

## Supporting information


**Figure S1.** Dot plot (A) and Feature plot (B) show the canonical marker genes for different cell clusters.

**Figure S2.** GSEA enrichment analysis in all PBMCs. (A‐B) GSEA enrichment plot showing pathways enriched in PD (A) or pSS (B) versus HCs. (C) Dot plots showed the scaled expression level and percentage of key genes involved in the pathways related to (A, B).

**Figure S3.** Multi‐volcano plot showing DEGs between PD or pSS patients with HCs in different cell clusters.

**Figure S4.** hdWGCNA analysis of T/NK cells. (A) The dendrogram identified four hdWGCNA modules in the T/NK cell cluster. (B) Dot plot for the enrichment of modules in different T/NK subclusters. (C) Function analysis for module 2 (M2) top 30 hub‐genes by Metascape. (D) Network plot showing top hub genes for M2. (E) Dot plots showed the average log_2_FC (avg_logFC) of key genes involved in the GO terms related to (C).

**Figure S5.** hdWGCNA analysis of myeloid cells. (A) The dendrogram identified four hdWGCNA modules in the myeloid cell cluster. (B) Dot plot for the enrichment of modules in different myeloid subclusters. (C) Function analysis for module 7 (M7) top 30 hub‐genes by Metascape. (D) Network plot showing top hub genes for M7. (E) Dot plots showed the average log_2_FC (avg_logFC) of key genes involved in the GO terms related to (C).

**Figure S6.** hdWGCNA analysis of B cells. (A) The dendrogram identified four hdWGCNA modules in the B cell cluster. (B) Dot plot for the enrichment of modules in different B cell subclusters. (C) Function analysis for module 1 (M1) top 30 hub‐genes by Metascape. (D) Network plot showing top hub genes for M1. (E) Dot plots showed the average log_2_FC (avg_logFC) of key genes involved in the GO terms related to (C).

**Figure S7.** Network of the potential signalling pathways between the ligand IL‐7 and its predicted target genes.

**Figure S8.** Dot plot (A) and heatmap plot (B) show the canonical marker genes for MDSCs.

**Table S1.** Information of samples used in this study.

**Table S2.** Cell number statistics of different cell clusters.

**Table S3.** DEGs between PD/pSS with HC in PBMCs.

**Table S4.** KEGG pathway enrichment of DEGs between PD/pSS with HC in PBMCs.

**Table S5.** Differentially expressed marker genes between PD/pSS and HC in subclusters of T/NK, myeloid and B cells.

**Table S6.** KEGG pathway enrichment of differentially expressed marker genes between PD/pSS with HC in subclusters of T/NK, myeloid cells and B cells.

**Table S7.** Cell number statistics in T/NK cells subclusters.

**Table S8.** Cell number statistics in myeloid cells subclusters.

**Table S9.** Cell number statistics in B cells subclusters.

## Data Availability

The datasets of single‐cell sequencing analysed during the current study are publicly available in the NCBI and NGDC.

## References

[1] Global Burden of Disease Study , “Global, Regional, and National Incidence, Prevalence, and Years Lived With Disability for 301 Acute and Chronic Diseases and Injuries in 188 Countries, 1990–2013: A Systematic Analysis for the Global Burden of Disease Study 2013,” Lancet 386, no. 9995 (2015): 743–800, 10.1016/S0140-6736(15)60692-4. 26063472 PMC4561509

[2] M. M. Prado , N. Figueiredo , A. L. Pimenta , et al., “Recent Updates on Microbial Biofilms in Periodontitis: An Analysis of In Vitro Biofilm Models,” Advances in Experimental Medicine and Biology 1373 (2022): 159–174, 10.1007/978-3-030-96881-6_8. 35612797

[3] T. Kwon , I. B. Lamster , and L. Levin , “Current Concepts in the Management of Periodontitis,” International Dental Journal 71, no. 6 (2021): 462–476, 10.1111/idj.12630. 34839889 PMC9275292

[4] B. A. Fisher , R. Jonsson , T. Daniels , et al., “Standardisation of Labial Salivary Gland Histopathology in Clinical Trials in Primary Sjögren's Syndrome,” Annals of the Rheumatic Diseases 76, no. 7 (2017): 1161–1168, 10.1136/annrheumdis-2016-210448. 27965259 PMC5530351

[5] S. Negrini , G. Emmi , M. Greco , et al., “Sjogren's Syndrome: A Systemic Autoimmune Disease,” Clinical and Experimental Medicine 22, no. 1 (2022): 9–25, 10.1007/s10238-021-00728-6. 34100160 PMC8863725

[6] G. E. Thorlacius , A. Bjork , and M. Wahren‐Herlenius , “Genetics and Epigenetics of Primary Sjogren Syndrome: Implications for Future Therapies,” Nature Reviews Rheumatology 19, no. 5 (2023): 288–306, 10.1038/s41584-023-00932-6. 36914790 PMC10010657

[7] M. G. Piccioni , L. Merlino , M. Deroma , et al., “The Impact of Primary Sjogren's Syndrome on Female Sexual Function,” Minerva Ginecologica 72, no. 1 (2020): 50–54, 10.23736/S0026-4784.20.04494-9. 32153164

[8] H. S. Choudhry , S. Hosseini , H. S. Choudhry , M. Fatahzadeh , R. Khianey , and M. H. Dastjerdi , “Updates in Diagnostics, Treatments, and Correlations Between Oral and Ocular Manifestations of Sjogren's Syndrome,” Ocular Surface 26 (2022): 75–87, 10.1016/j.jtos.2022.08.001. 35961534

[9] D. L. Cartee , S. Maker , D. Dalonges , and M. C. Manski , “Sjogren's Syndrome: Oral Manifestations and Treatment, a Dental Perspective,” Journal of Dental Hygiene 89, no. 6 (2015): 365–371. 26684993

[10] M. P. Najera , I. al‐Hashimi , J. M. Plemons , et al., “Prevalence of Periodontal Disease in Patients With Sjogren's Syndrome,” Oral Surgery, Oral Medicine, Oral Pathology, Oral Radiology, and Endodontology 83, no. 4 (1997): 453–457, 10.1016/s1079-2104(97)90144-x. 9127376

[11] C. Y. Lin , C. F. Tseng , J. M. Liu , et al., “Association Between Periodontal Disease and Subsequent Sjogren's Syndrome: A Nationwide Population‐Based Cohort Study,” International Journal of Environmental Research and Public Health 16, no. 5 (2019): 771, 10.3390/ijerph16050771. 30832451 PMC6427323

[12] M. C. Lu , C. H. Jheng , T. Y. Tsai , M. Koo , and N. S. Lai , “Increased Dental Visits in Patients Prior to Diagnosis of Primary Sjogren's Syndrome: A Population‐Based Study in Taiwan,” Rheumatology International 34, no. 11 (2014): 1555–1561, 10.1007/s00296-014-3003-5. 24706034

[13] K. Conde , C. O. Guelngar , M. C. Barry , H. G. Atakla , A. Mohamed , and F. A. Cisse , “Sjögren's Syndrome in Children: About 15 Cases in Guinea Conakry,” European Journal of Medical Research 26, no. 1 (2021): 66, 10.1186/s40001-021-00534-6. 34215328 PMC8252240

[14] B. Yang , X. Pang , J. Guan , et al., “The Association of Periodontal Diseases and Sjogren's Syndrome: A Systematic Review and Meta‐Analysis,” Frontiers in Medicine 9 (2022): 904638, 10.3389/fmed.2022.904638. 36687426 PMC9851607

[15] Y. Zhou and Z. Liu , “Saliva Biomarkers in Oral Disease,” Clinica Chimica Acta 548 (2023): 117503, 10.1016/j.cca.2023.117503. 37536520

[16] J. G. Caton , G. Armitage , T. Berglundh , et al., “A New Classification Scheme for Periodontal and Peri‐Implant Diseases and Conditions—Introduction and Key Changes From the 1999 Classification,” Journal of Periodontology 89, no. S1 (2018): S1–S8, 10.1002/JPER.18-0157. 29926946

[17] M. M. Azuma , R. O. Samuel , J. E. Gomes‐Filho , E. Dezan‐Junior , and L. T. Cintra , “The Role of IL‐6 on Apical Periodontitis: A Systematic Review,” International Endodontic Journal 47, no. 7 (2014): 615–621, 10.1111/iej.12196. 24224782

[18] L. A. Moreno‐Quispe , J. Serrano , L. Virto , et al., “Association of Salivary Inflammatory Biomarkers With Primary Sjogren's Syndrome,” Journal of Oral Pathology & Medicine 49, no. 9 (2020): 940–947, 10.1111/jop.13070. 32538490

[19] L. Nibali , S. Fedele , F. D'Aiuto , and N. Donos , “Interleukin‐6 in Oral Diseases: A Review,” Oral Diseases 18, no. 3 (2012): 236–243, 10.1111/j.1601-0825.2011.01867.x. 22050374

[20] G. M. Verstappen , O. B. J. Corneth , H. Bootsma , and F. G. M. Kroese , “Th17 Cells in Primary Sjogren's Syndrome: Pathogenicity and Plasticity,” Journal of Autoimmunity 87 (2018): 16–25, 10.1016/j.jaut.2017.11.003. 29191572

[21] K. Bunte and T. Beikler , “Th17 Cells and the IL‐23/IL‐17 Axis in the Pathogenesis of Periodontitis and Immune‐Mediated Inflammatory Diseases,” International Journal of Molecular Sciences 20, no. 14 (2019): 3394, 10.3390/ijms20143394. 31295952 PMC6679067

[22] L. Melguizo‐Rodriguez , V. J. Costela‐Ruiz , F. J. Manzano‐Moreno , C. Ruiz , and R. Illescas‐Montes , “Salivary Biomarkers and Their Application in the Diagnosis and Monitoring of the Most Common Oral Pathologies,” International Journal of Molecular Sciences 21, no. 14 (2020): 5173, 10.3390/ijms21145173. 32708341 PMC7403990

[23] S. Longhino , L. G. Chatzis , R. Dal Pozzolo , et al., “Sjogren's Syndrome: One Year in Review 2023,” Clinical and Experimental Rheumatology 41, no. 12 (2023): 2343–2356, 10.55563/clinexprheumatol/255qsx. 38149515

[24] G. M. Verstappen , F. G. M. Kroese , and H. Bootsma , “T Cells in Primary Sjogren's Syndrome: Targets for Early Intervention,” Rheumatology 60, no. 7 (2021): 3088–3098, 10.1093/rheumatology/kez004. 30770920 PMC8516500

[25] T. Berglundh , M. Donati , and N. Zitzmann , “B Cells in Periodontitis: Friends or Enemies?,” Periodontology 2000 45 (2007): 51–66, 10.1111/j.1600-0757.2007.00223.x. 17850448

[26] M. Kebschull , P. Guarnieri , R. T. Demmer , A. L. Boulesteix , P. Pavlidis , and P. N. Papapanou , “Molecular Differences Between Chronic and Aggressive Periodontitis,” Journal of Dental Research 92, no. 12 (2013): 1081–1088, 10.1177/0022034513506011. 24122488 PMC3834653

[27] J. O. Pers , F. d'Arbonneau , V. Devauchelle‐Pensec , A. Saraux , Y. L. Pennec , and P. Youinou , “Is Periodontal Disease Mediated by Salivary BAFF in Sjogren's Syndrome?,” Arthritis & Rheumatism 52, no. 8 (2005): 2411–2414, 10.1002/art.21205. 16052575

[28] L. Wen and F. Tang , “Recent Advances in Single‐Cell Sequencing Technologies,” Precision Clinical Medicine 5, no. 1 (2022): pbac002, 10.1093/pcmedi/pbac002. 35821681 PMC9267251

[29] X. Hong , S. Meng , D. Tang , et al., “Single‐Cell RNA Sequencing Reveals the Expansion of Cytotoxic CD4(+) T Lymphocytes and a Landscape of Immune Cells in Primary Sjogren's Syndrome,” Frontiers in Immunology 11 (2020): 594658, 10.3389/fimmu.2020.594658. 33603736 PMC7884617

[30] Y. Wang , X. Xie , C. Zhang , et al., “Rheumatoid Arthritis, Systemic Lupus Erythematosus and Primary Sjogren's Syndrome Shared Megakaryocyte Expansion in Peripheral Blood,” Annals of the Rheumatic Diseases 81, no. 3 (2022): 379–385, 10.1136/annrheumdis-2021-220066. 34462261 PMC8862024

[31] J. Kang , H. Lee , J. Y. Joo , et al., “Comparison of Genetic and Epigenetic Profiles of Periodontitis According to the Presence of Type 2 Diabetes,” MedComm 5, no. 7 (2024): e620, 10.1002/mco2.620. 38903536 PMC11187843

[32] H. Lee , J. Y. Joo , D. H. Sohn , et al., “Single‐Cell RNA Sequencing Reveals Rebalancing of Immunological Response in Patients With Periodontitis After Non‐Surgical Periodontal Therapy,” Journal of Translational Medicine 20, no. 1 (2022): 504, 10.1186/s12967-022-03702-2. 36329504 PMC9635198

[33] G. X. Zheng , J. M. Terry , P. Belgrader , et al., “Massively Parallel Digital Transcriptional Profiling of Single Cells,” Nature Communications 8 (2017): 14049, 10.1038/ncomms14049. PMC5241818 28091601

[34] Y. Hao , T. Stuart , M. H. Kowalski , et al., “Dictionary Learning for Integrative, Multimodal and Scalable Single‐Cell Analysis,” Nature Biotechnology 42, no. 2 (2024): 293–304, 10.1038/s41587-023-01767-y. PMC10928517 37231261

[35] T. Wu , E. Hu , S. Xu , et al., “clusterProfiler 4.0: A Universal Enrichment Tool for Interpreting Omics Data,” Innovations 2, no. 3 (2021): 100141, 10.1016/j.xinn.2021.100141. PMC8454663 34557778

[36] S. Morabito , F. Reese , N. Rahimzadeh , E. Miyoshi , and V. Swarup , “hdWGCNA Identifies Co‐Expression Networks in High‐Dimensional Transcriptomics Data,” Cell Reports Methods 3, no. 6 (2023): 100498, 10.1016/j.crmeth.2023.100498. 37426759 PMC10326379

[37] R. Browaeys , W. Saelens , and Y. Saeys , “NicheNet: Modeling Intercellular Communication by Linking Ligands to Target Genes,” Nature Methods 17, no. 2 (2020): 159–162, 10.1038/s41592-019-0667-5. 31819264

[38] J. J. Pen , B. De Keersmaecker , S. K. Maenhout , et al., “Modulation of Regulatory T Cell Function by Monocyte‐Derived Dendritic Cells Matured Through Electroporation With mRNA Encoding CD40 Ligand, Constitutively Active TLR4, and CD70,” Journal of Immunology 191, no. 4 (2013): 1976–1983, 10.4049/jimmunol.1201008. 23842750

[39] U. Hainz , P. Obexer , C. Winkler , et al., “Monocyte‐Mediated T‐Cell Suppression and Augmented Monocyte Tryptophan Catabolism After Human Hematopoietic Stem‐Cell Transplantation,” Blood 105, no. 10 (2005): 4127–4134, 10.1182/blood-2004-05-1726. 15677560 PMC1895091

[40] D. I. Gabrilovich and S. Nagaraj , “Myeloid‐Derived Suppressor Cells as Regulators of the Immune System,” Nature Reviews. Immunology 9, no. 3 (2009): 162–174, 10.1038/nri2506. PMC2828349 19197294

[41] K. Takada and S. C. Jameson , “Naive T Cell Homeostasis: From Awareness of Space to a Sense of Place,” Nature Reviews. Immunology 9, no. 12 (2009): 823–832, 10.1038/nri2657. 19935802

[42] L. M. McLane , M. S. Abdel‐Hakeem , and E. J. Wherry , “CD8 T Cell Exhaustion During Chronic Viral Infection and Cancer,” Annual Review of Immunology 37 (2019): 457–495, 10.1146/annurev-immunol-041015-055318. 30676822

[43] E. J. Wherry and M. Kurachi , “Molecular and Cellular Insights Into T Cell Exhaustion,” Nature Reviews. Immunology 15, no. 8 (2015): 486–499, 10.1038/nri3862. PMC4889009 26205583

[44] E. J. Wherry , “T Cell Exhaustion,” Nature Immunology 12, no. 6 (2011): 492–499, 10.1038/ni.2035. 21739672

[45] N. Medara , J. C. Lenzo , K. A. Walsh , et al., “Peripheral Memory T‐Cell Profile Is Modified in Patients Undergoing Periodontal Management,” Journal of Clinical Periodontology 48, no. 2 (2021): 249–262, 10.1111/jcpe.13399. 33131124

[46] I. Kudryavtsev , S. Benevolenskaya , M. Serebriakova , et al., “Circulating CD8^+^ T Cell Subsets in Primary Sjogren's Syndrome,” Biomedicine 11, no. 10 (2023): 2778, 10.3390/biomedicines11102778. PMC10604770 37893153

[47] J. Demoersman , P. Pochard , C. Framery , et al., “B Cell Subset Distribution Is Altered in Patients With Severe Periodontitis,” PLoS One 13, no. 2 (2018): e0192986, 10.1371/journal.pone.0192986. 29447240 PMC5814041

[48] O. A. Gonzalez , M. J. Novak , S. Kirakodu , et al., “Comparative Analysis of Gingival Tissue Antigen Presentation Pathways in Ageing and Periodontitis,” Journal of Clinical Periodontology 41, no. 4 (2014): 327–339, 10.1111/jcpe.12212. 24304139 PMC3951170

[49] J. Sprent and C. D. Surh , “Normal T Cell Homeostasis: The Conversion of Naive Cells Into Memory‐Phenotype Cells,” Nature Immunology 12, no. 6 (2011): 478–484, 10.1038/ni.2018. 21739670 PMC3434123

[50] T. Kawabe , J. Yi , and J. Sprent , “Homeostasis of Naive and Memory T Lymphocytes,” Cold Spring Harbor Perspectives in Biology 13, no. 9 (2021): a037879, 10.1101/cshperspect.a037879. 33753403 PMC8411951

[51] M. J. Palmer , V. S. Mahajan , J. Chen , D. J. Irvine , and D. A. Lauffenburger , “Signaling Thresholds Govern Heterogeneity in IL‐7‐Receptor‐Mediated Responses of Naïve CD8+ T Cells,” Immunology and Cell Biology 89, no. 5 (2011): 581–594, 10.1038/icb.2011.5. 21339767 PMC3342499

[52] E. Desvaux , P. Hemon , P. Soret , et al., “High‐Content Multimodal Analysis Supports the IL‐7/IL‐7 Receptor Axis as a Relevant Therapeutic Target in Primary Sjogren's Syndrome,” Journal of Autoimmunity 149 (2023): 103147, 10.1016/j.jaut.2023.103147. 38114349

[53] Y. Liang , Z. Zhang , J. Li , W. Luo , T. Jiang , and Z. Yang , “Association Between IL‐7 and Primary Sjogren's Syndrome: A Single‐Center Study and a Systematic Scoping Review,” International Immunopharmacology 108 (2022): 108758, 10.1016/j.intimp.2022.108758. 35405597

[54] J. H. Cho , H. O. Kim , C. D. Surh , and J. Sprent , “T Cell Receptor‐Dependent Regulation of Lipid Rafts Controls Naive CD8+ T Cell Homeostasis,” Immunity 32, no. 2 (2010): 214–226, 10.1016/j.immuni.2009.11.014. 20137986 PMC2830358

[55] J. Verwilghen , G. H. Kingsley , J. L. Ceuppens , and G. S. Panayi , “Inhibition of Synovial Fluid T Cell Proliferation by Anti‐CD5 Monoclonal Antibodies. A Potential Mechanism for Their Immunotherapeutic Action In Vivo,” Arthritis and Rheumatism 35, no. 12 (1992): 1445–1451, 10.1002/art.1780351207. 1282008

[56] T. So and N. Ishii , “The TNF‐TNFR Family of co‐Signal Molecules,” Advances in Experimental Medicine and Biology 1189 (2019): 53–84, 10.1007/978-981-32-9717-3_3. 31758531

[57] C. J. Nile , S. Sherrabeh , G. Ramage , and D. F. Lappin , “Comparison of Circulating Tumour Necrosis Factor Superfamily Cytokines in Periodontitis Patients Undergoing Supportive Therapy: A Case‐Controlled Cross‐Sectional Study Comparing Smokers and Non‐Smokers in Health and Disease,” Journal of Clinical Periodontology 40, no. 9 (2013): 875–882, 10.1111/jcpe.12134. 23919740

[58] S. L. Teitelbaum , “Bone Resorption by Osteoclasts,” Science 289, no. 5484 (2000): 1504–1508, 10.1126/science.289.5484.1504. 10968780

[59] S. Nakae , H. Suto , M. Iikura , et al., “Mast Cells Enhance T Cell Activation: Importance of Mast Cell Costimulatory Molecules and Secreted TNF,” Journal of Immunology 176, no. 4 (2006): 2238–2248, 10.4049/jimmunol.176.4.2238. 16455980

[60] J. Kashiwakura , H. Yokoi , H. Saito , and Y. Okayama , “T Cell Proliferation by Direct Cross‐Talk Between OX40 Ligand on Human Mast Cells and OX40 on Human T Cells: Comparison of Gene Expression Profiles Between Human Tonsillar and Lung‐Cultured Mast Cells,” Journal of Immunology 173, no. 8 (2004): 5247–5257, 10.4049/jimmunol.173.8.5247. 15470070

[61] Y. J. Yeap , N. Kandiah , D. Nizetic , and K. L. Lim , “BACE2: A Promising Neuroprotective Candidate for Alzheimer's Disease,” Journal of Alzheimer's Disease 94, no. s1 (2023): S159–S171, 10.3233/JAD-220867. PMC10473127 36463454

[62] M. R. Nepal , S. Shah , and K. T. Kang , “Dual Roles of Myeloid‐Derived Suppressor Cells in Various Diseases: A Review,” Archives of Pharmacal Research 47, no. 7 (2024): 597–616, 10.1007/s12272-024-01504-2. 39008186

[63] Y. Miyahara , S. Takano , K. Sogawa , et al., “Prosaposin, Tumor‐Secreted Protein, Promotes Pancreatic Cancer Progression by Decreasing Tumor‐Infiltrating Lymphocytes,” Cancer Science 113, no. 8 (2022): 2548–2559, 10.1111/cas.15444. 35633503 PMC9357616

[64] J. Tinklepaugh , B. M. Smith , E. Hanlon , C. Zubieta , F. Bou‐Abdallah , and R. P. Doyle , “Exploring the Multiligand Binding Specificity of Saposin B Reveals Two Binding Sites,” ACS Omega 2, no. 10 (2017): 7141–7145, 10.1021/acsomega.7b01334. 29104953 PMC5664142

[65] B. Schiemann , J. L. Gommerman , K. Vora , et al., “An Essential Role for BAFF in the Normal Development of B Cells Through a BCMA‐Independent Pathway,” Science 293, no. 5537 (2001): 2111–2114, 10.1126/science.1061964. 11509691

[66] M. T. Abo‐Elfadl , A. M. Gamal‐Eldeen , M. F. Ismail , and N. N. Shahin , “Silencing of the Cytokine Receptor TNFRSF13B: A New Therapeutic Target for Triple‐Negative Breast Cancer,” Cytokine 125 (2020): 154790, 10.1016/j.cyto.2019.154790. 31400636

[67] C. W. Cutler and R. Jotwani , “Antigen‐Presentation and the Role of Dendritic Cells in Periodontitis,” Periodontology 2000 35 (2004): 135–157, 10.1111/j.0906-6713.2004.003560.x. 15107061

[68] X. Liu , H. L. Ong , and I. Ambudkar , “TRP Channel Involvement in Salivary Glands‐Some Good, Some Bad,” Cells 7, no. 7 (2018): 74, 10.3390/cells7070074. 29997338 PMC6070825

[69] Q. Li , M. S. Valerio , and K. L. Kirkwood , “MAPK Usage in Periodontal Disease Progression,” Journal of Signal Transduction 2012 (2012): 308943, 10.1155/2012/308943. 22315682 PMC3270463

[70] N. Cao , H. Shi , C. Chen , L. Zheng , and C. Yu , “Inhibition of the TLR9‐Dependent p38 MAPK Signaling Pathway Improves the Pathogenesis of Primary Sjogren's Syndrome in the NOD/Ltj Mouse,” Journal of Biological Regulators and Homeostatic Agents 35, no. 3 (2021): 1103–1108, 10.23812/21-154-L. 34034463

[71] D. M. Salerno , J. S. Tront , B. Hoffman , and D. A. Liebermann , “Gadd45a and Gadd45b Modulate Innate Immune Functions of Granulocytes and Macrophages by Differential Regulation of p38 and JNK Signaling,” Journal of Cellular Physiology 227, no. 11 (2012): 3613–3620, 10.1002/jcp.24067. 22307729

[72] X. Liu , Y. Wang , H. Lu , et al., “Genome‐Wide Analysis Identifies NR4A1 as a Key Mediator of T Cell Dysfunction,” Nature 567, no. 7749 (2019): 525–529, 10.1038/s41586-019-0979-8. 30814730 PMC6507425

