# Supplementary figures and images for: Featured Immune Characteristics of Periodontitis and Primary Sjögren's Syndrome Revealed by Single‐Cell Transcriptome Analyses
Source: J Cell Mol Med. 2025 Jul 22;29(14):e70713. doi: 10.1111/jcmm.70713 (PMC12283242; doi:10.1111/jcmm.70713)

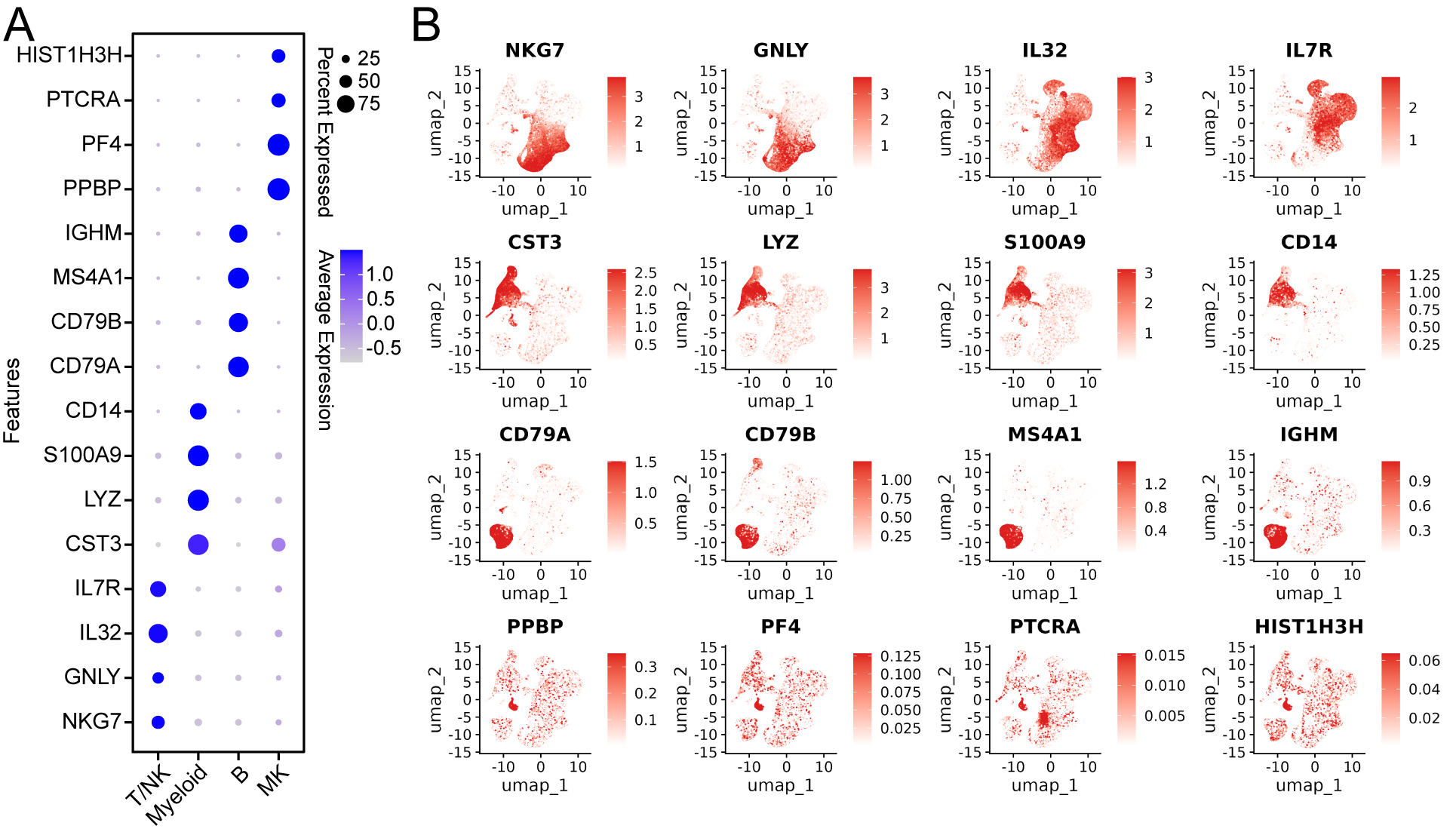

Supplement: Supplementary file 1 — Figure S1. Dot plot (A) and Feature plot (B) show the canonical marker genes for different cell clusters. [file JCMM-29-e70713-s001.tif]

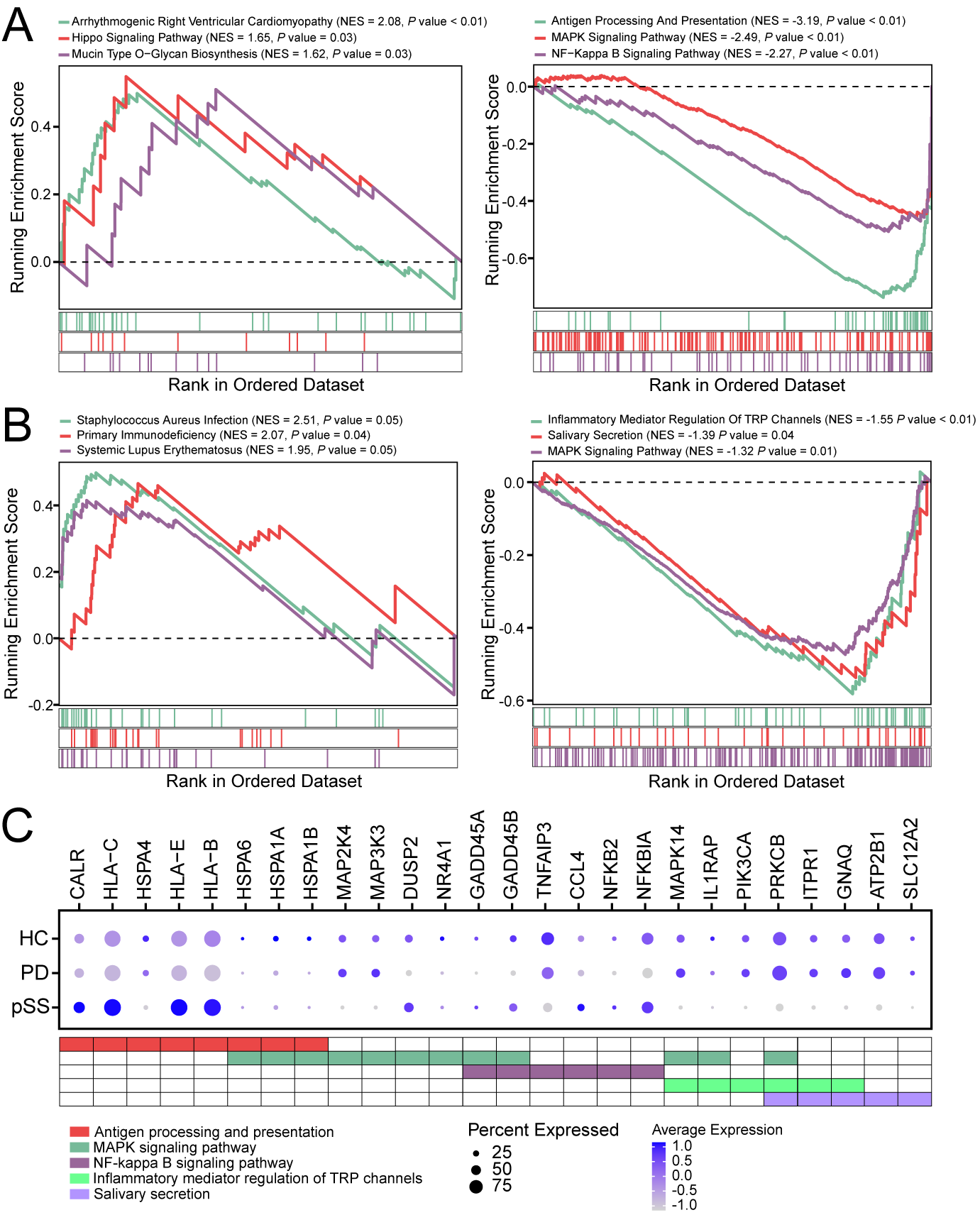

Supplement: Supplementary file 2 — Figure S2. GSEA enrichment analysis in all PBMCs. (A‐B) GSEA enrichment plot showing pathways enriched in PD (A) or pSS (B) versus HCs. (C) Dot plots showed the scaled expression level and percentage of key genes involved in the pathways related to (A, B). [file JCMM-29-e70713-s002.tif]

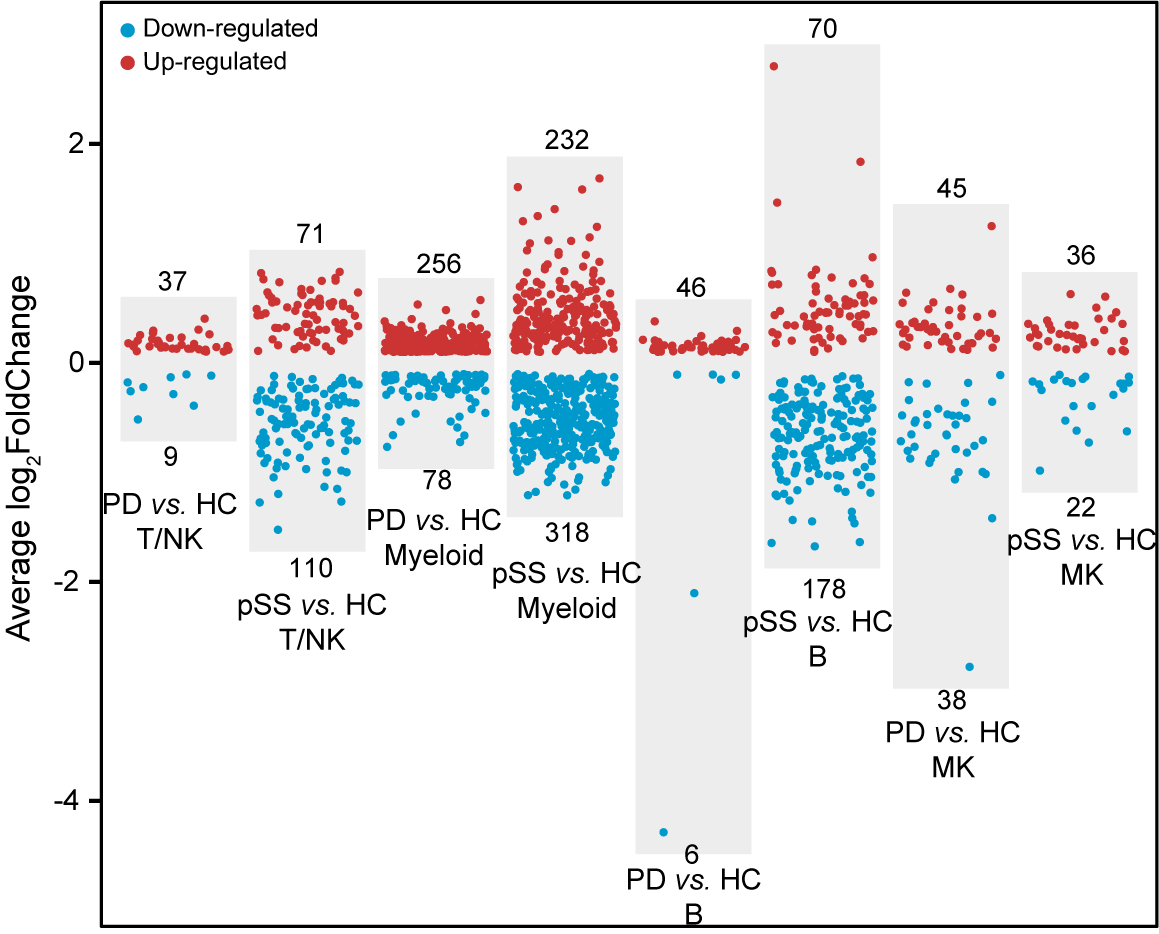

Supplement: Supplementary file 3 — Figure S3. Multi‐volcano plot showing DEGs between PD or pSS patients with HCs in different cell clusters. [file JCMM-29-e70713-s013.tif]

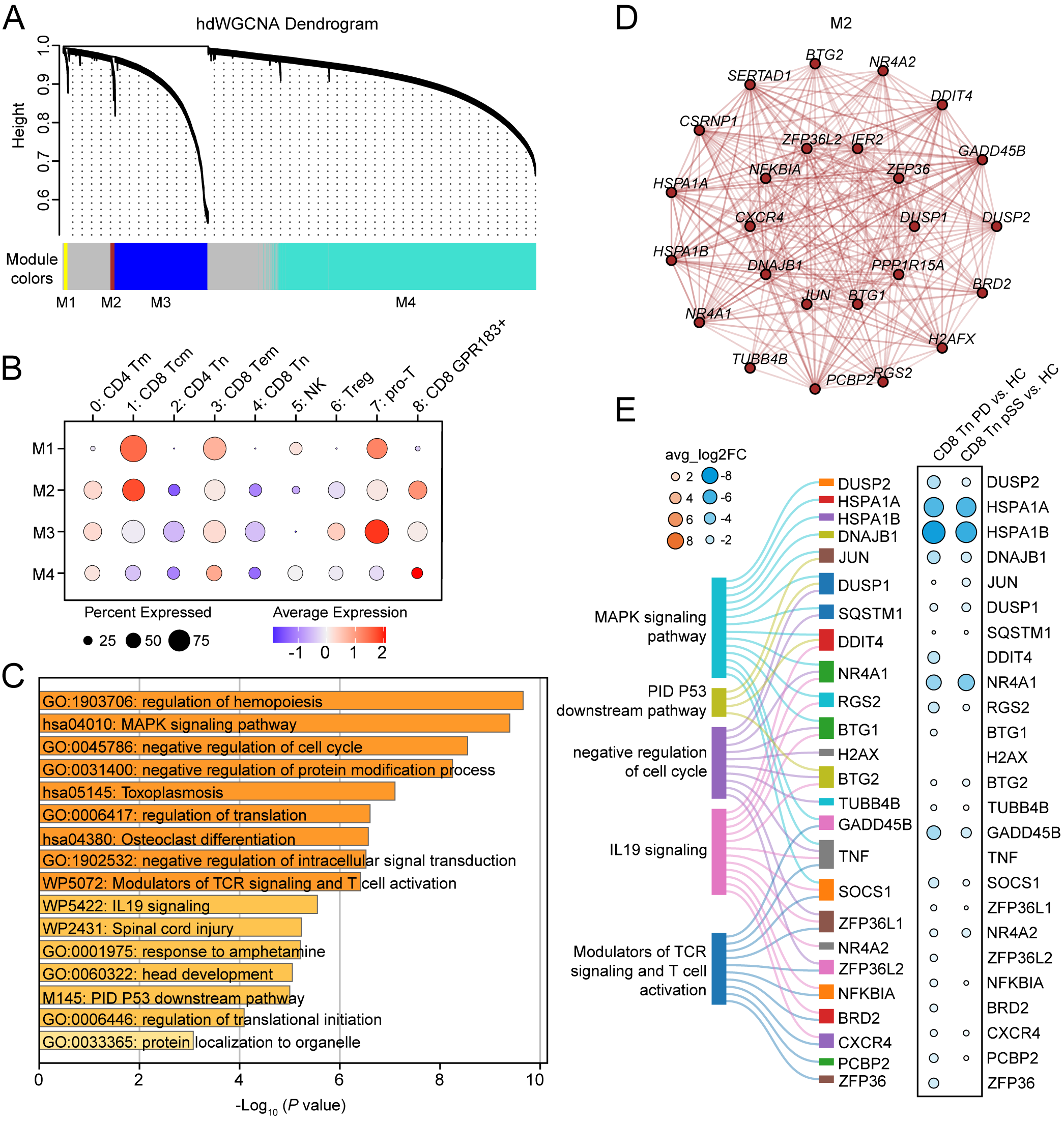

Supplement: Supplementary file 4 — Figure S4. hdWGCNA analysis of T/NK cells. (A) The dendrogram identified four hdWGCNA modules in the T/NK cell cluster. (B) Dot plot for the enrichment of modules in different T/NK subclusters. (C) Function analysis for module 2 (M2) top 30 hub‐genes by Metascape. (D) Network plot showing top hub genes for M2. (E) Dot plots showed the average log2FC (avg_logFC) of key genes involved in the GO terms related to (C). [file JCMM-29-e70713-s016.tif]

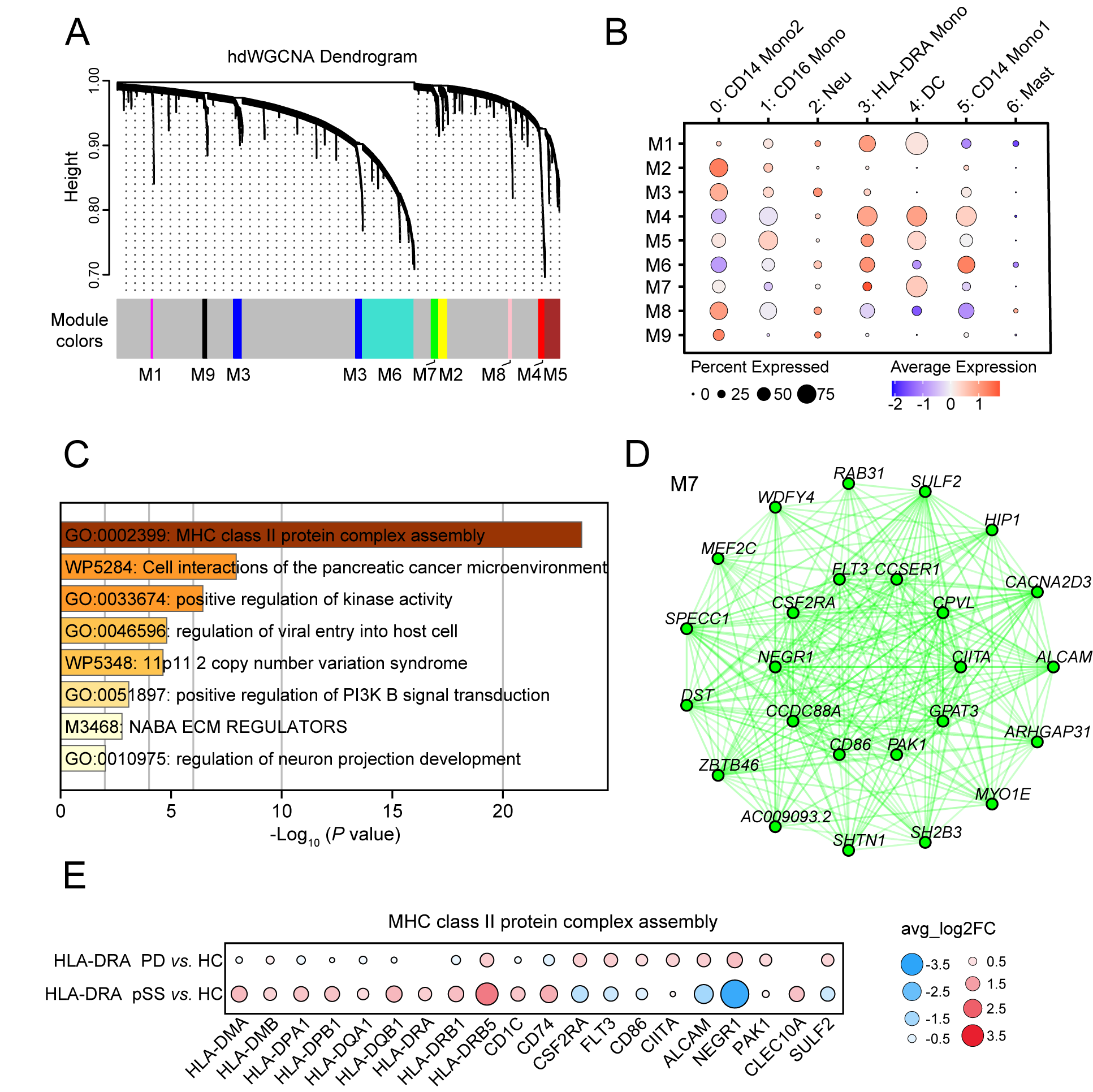

Supplement: Supplementary file 5 — Figure S5. hdWGCNA analysis of myeloid cells. (A) The dendrogram identified four hdWGCNA modules in the myeloid cell cluster. (B) Dot plot for the enrichment of modules in different myeloid subclusters. (C) Function analysis for module 7 (M7) top 30 hub‐genes by Metascape. (D) Network plot showing top hub genes for M7. (E) Dot plots showed the average log2FC (avg_logFC) of key genes involved in the GO terms related to (C). [file JCMM-29-e70713-s005.tif]

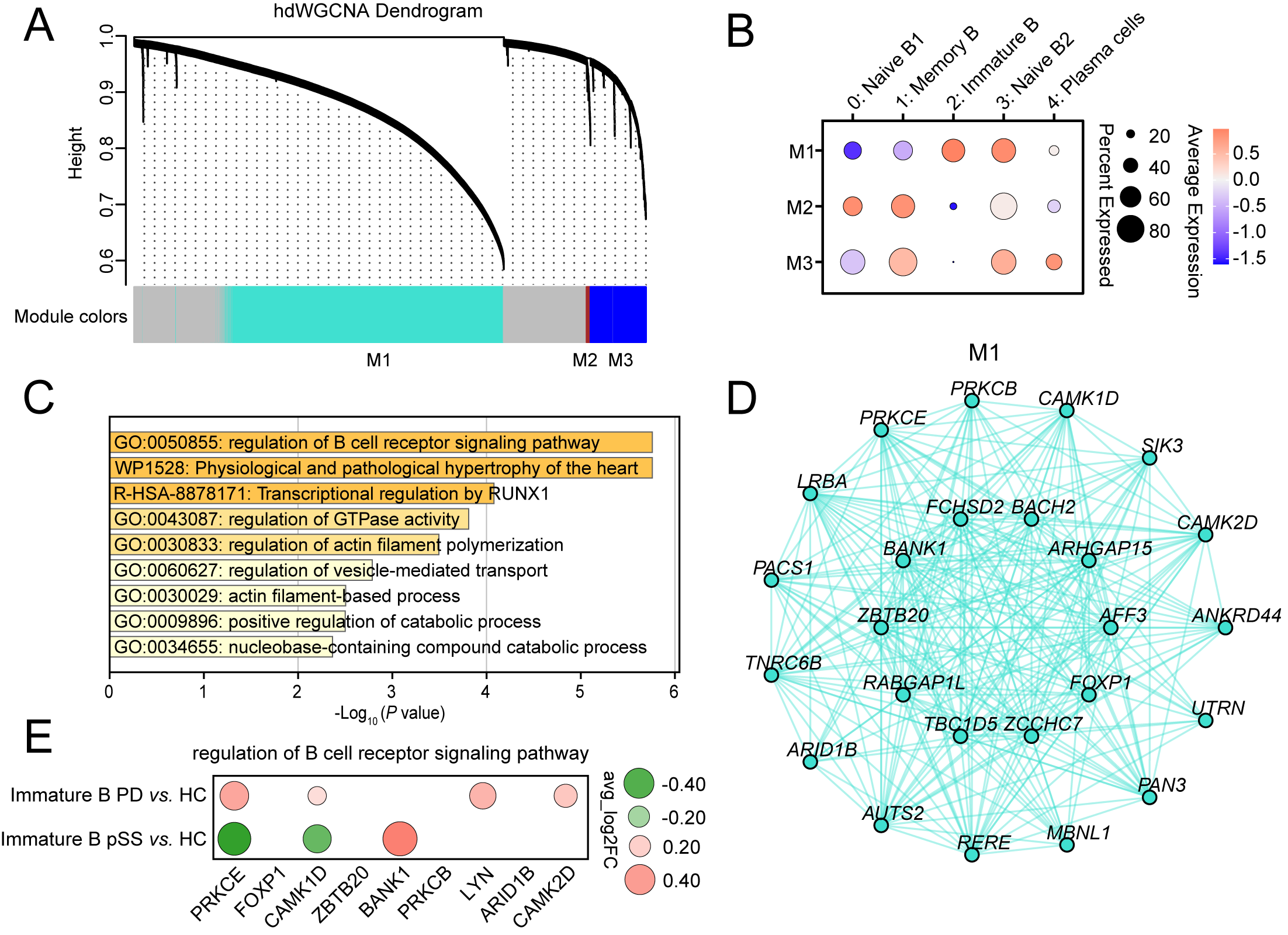

Supplement: Supplementary file 6 — Figure S6. hdWGCNA analysis of B cells. (A) The dendrogram identified four hdWGCNA modules in the B cell cluster. (B) Dot plot for the enrichment of modules in different B cell subclusters. (C) Function analysis for module 1 (M1) top 30 hub‐genes by Metascape. (D) Network plot showing top hub genes for M1. (E) Dot plots showed the average log2FC (avg_logFC) of key genes involved in the GO terms related to (C). [file JCMM-29-e70713-s007.tif]

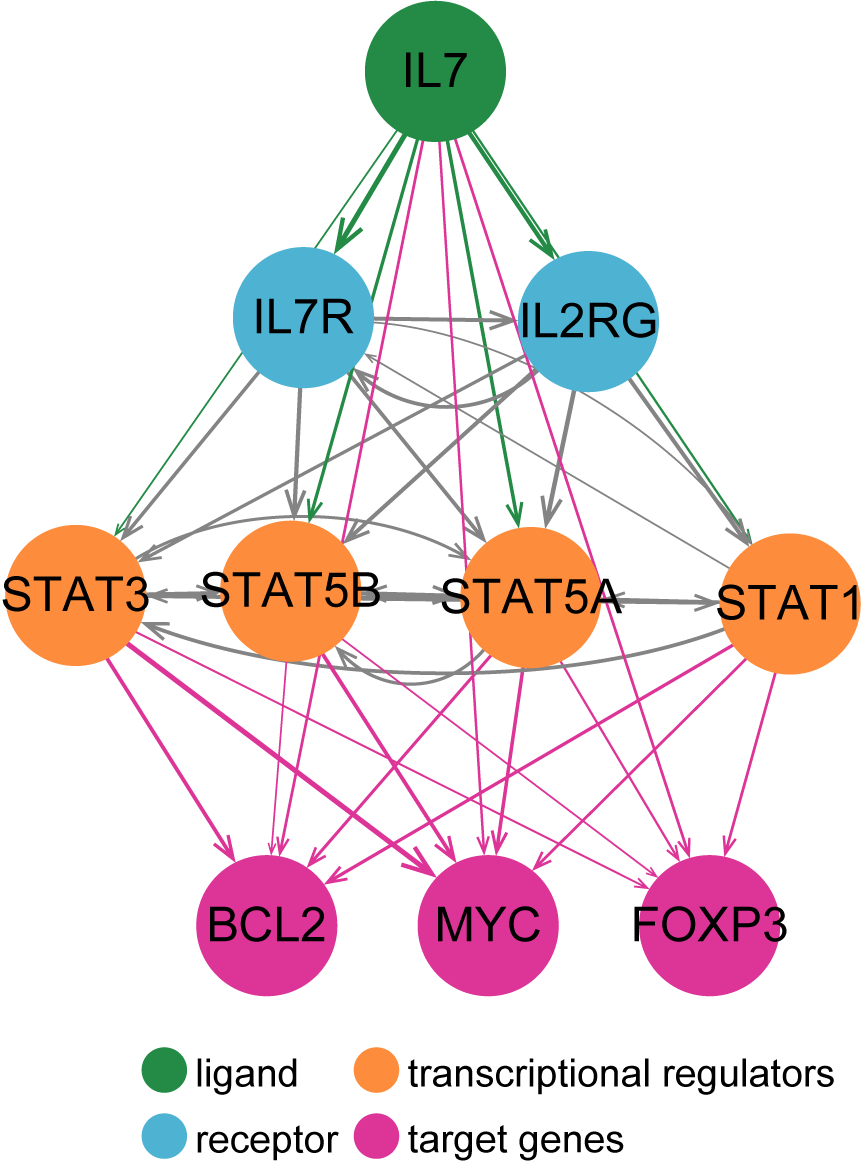

Supplement: Supplementary file 7 — Figure S7. Network of the potential signalling pathways between the ligand IL‐7 and its predicted target genes. [file JCMM-29-e70713-s017.tif]

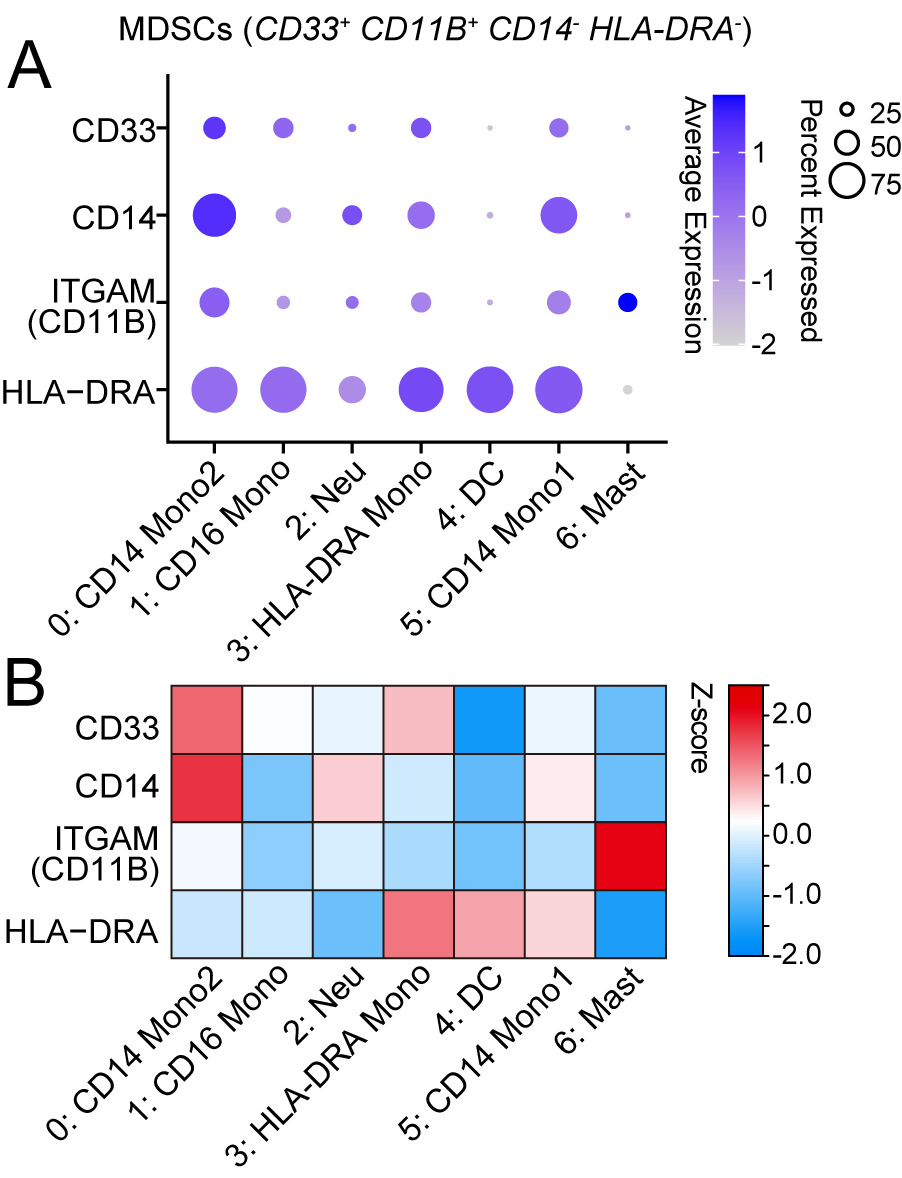

Supplement: Supplementary file 8 — Figure S8. Dot plot (A) and heatmap plot (B) show the canonical marker genes for MDSCs. [file JCMM-29-e70713-s009.tif]
